# Antibiotics and phages drive region-specific diversity of OmpK36 in *Klebsiella pneumoniae*

**DOI:** 10.1128/mbio.01343-25

**Published:** 2025-08-18

**Authors:** Guilhem Royer, Raphaël Laurenceau, Nicolas Cabanel, Benjamin Bardiaux, Karol Melissa Cerdas-Mejías, David Bikard, Isabelle Rosinski-Chupin, Philippe Glaser

**Affiliations:** 1 Institut Pasteur, Université Paris Cité, CNRS UMR6047, Ecology and Evolution of Antibiotic Resistance Unit 555089, Paris, France; 2 Unité de Bactériologie, Département de Prévention, Diagnostic et Traitement des Infections, AP-HP, Hôpital Henri Mondor, Créteil, France; 3 EA 7380 Dynamyc, EnvA, UPEC, Université Paris-Est Créteil, Créteil, France; 4 Institut Pasteur, Université de Paris Cité, CNRS UMR3525, Synthetic Biology Unit 27058, Paris, France; 5 Institut Pasteur, Université Paris Cité, CNRS UMR3528, Structural Bioinformatics Unit 27058, Paris, France; 6 Institut Pasteur, Université Paris Cité, CNRS UMR3528, Bacterial Transmembrane Systems Unit 27058, Paris, France; National University of Singapore, Singapore, Singapore

**Keywords:** porins, genome analysis, bacteriophages, antibiotic resistance, OmpK36

## Abstract

The *Enterobacterales* outer membrane is impermeable to many antibiotics, but they can enter the cell via channel proteins, called porins. In *Klebsiella pneumoniae*, the porin OmpK36 is a major determinant of antibiotic resistance. Insertion of amino acids into the L3 constriction loop is frequently reported, leading to decreased channel size and reduced antibiotic susceptibility. While the involvement of the L3 loop in resistance is well-established, studies focused on a limited number of sequences, without considering any role for diversity outside L3. To fill this gap, we carried out a large-scale genomic comparison combined with experimental analysis. We analyzed 16,086 *K*. *pneumoniae* genomes to decipher the diversity of OmpK36 at the species level. We identified 385 protein variants classified into 7 main backbones, some associated with high-risk clones and, consequently, with major resistance classes. Comparison of predicted three-dimensional structures indicates variation in pore size depending on backbones. We constructed mutants carrying the main variants and compared their fitness and susceptibility to antibiotics and phages. Despite OmpK36 diversity and predicted pore radius differences, variations beyond the constriction loop do not seem to significantly impact antibiotic susceptibility and fitness. However, this diversity may have been partly shaped by phages targeting OmpK36, as evidenced by varying susceptibility to a T4-like phage depending on the backbones and the rapid acquisition of mutations in *ompK36* among initially susceptible mutants following phage exposure. These findings suggest that while antibiotic pressure selects for L3 loop variations, phage predation may be a key driver of broader OmpK36 diversity.

**IMPORTANCE:**

Porins are outer membrane proteins involved in the passive diffusion into the periplasm of substrates such as nutrients. They are also a major route for antibiotics. Mutations in the main porins of *K. pneumoniae*, OmpK35 and OmpK36, have been described and mainly lead to disruption of the former and a pore size reduction of the latter through amino acid insertion in the constriction loop. *ompK36* mutations are clinically significant, with a reduction in susceptibility to β-lactams, particularly when strains also produce a carbapenemase. However, little is known about the overall diversity of OmpK36. Through large-scale genome analysis and experimental work, we sought to decipher the selective pressures that shape the diversity of this protein. Our results suggest that antibiotics are not the main driver of OmpK36 diversity at the level of the entire protein sequence and that phages can instead rapidly select variations in the extracellular loops.

## INTRODUCTION

*Klebsiella pneumoniae* is a major bacterial pathogen and a critical threat due to its frequent multidrug resistance, particularly in healthcare settings (1–3). For β-lactams, resistance in *K. pneumoniae* is mainly driven by the acquisition of β-lactamase coding genes such as extended-spectrum β-lactamases (ESBL) and carbapenemases, often combined with mutations in the two major porin genes encoding OmpK35 (4) and OmpK36 (5), homologs of *Escherichia coli* OmpF and OmpC, respectively. These outer membrane proteins fold into a β-barrel with seven extracellular loops, along with another loop, L3, which extends inside the barrel and constricts the pore (5, 6). Mutations in porins play a pivotal role in the diffusion of β-lactams resistance particularly in healthcare settings (7). Moreover, OmpK36, as well as a few other porins, has been shown to be involved in plasmid mating pair stabilization and conjugation species specificity, with a significant impact on the distribution of resistance plasmids (8).

Truncations in *ompK35* are frequent in clinical isolates, especially in high-risk clones that have disseminated worldwide (9). *ompK35* deletion has no fitness cost *in vitro*, except in minimal media, and a limited cost *in vivo* (10, 11). Conversely, *ompK36* deletion is associated with a reduction in fitness under both conditions (10). This may explain the low frequency of deletion among clinical isolates (12) despite the reduced antibiotic susceptibility it confers (13, 14). On the other hand, mutations leading to amino acid insertions (mainly GD, TD, D, and SD) in the L3 constriction loop of Ompk36 have been described for more than 10 years (15, 16). These mutations may be viewed as a compromise as they confer almost the same resistance phenotype as complete deletion of the porin gene (11) while limiting the impact in terms of fitness and infection capacity (12). Truncations in *ompK35* combined with insertions in *ompK36* are frequent in carbapenemase-producing isolates and have been shown to enhance the resistance phenotype (11, 12) as in the ST258/512, a major pandemic multi-resistant lineage (9, 12).

Thanks to comprehensive studies combining genomic epidemiology and experimental data, we have now accumulated extensive knowledge on the impact of these mutations both on resistance and virulence. However, it was only examined through the prism of these mutations in L3, without considering the whole diversity of OmpK36. In this context, we aimed to decipher the diversity of OmpK36 at the *K. pneumoniae* subsp. *pneumoniae* level through both *in silico* and experimental approaches. Our objective was to identify clues about the selective pressure acting on OmpK36, shaping its diversity.

## RESULTS

### High diversity of OmpK36 at the species level, with seven main backbones and very frequent variants

**Fig 1:**
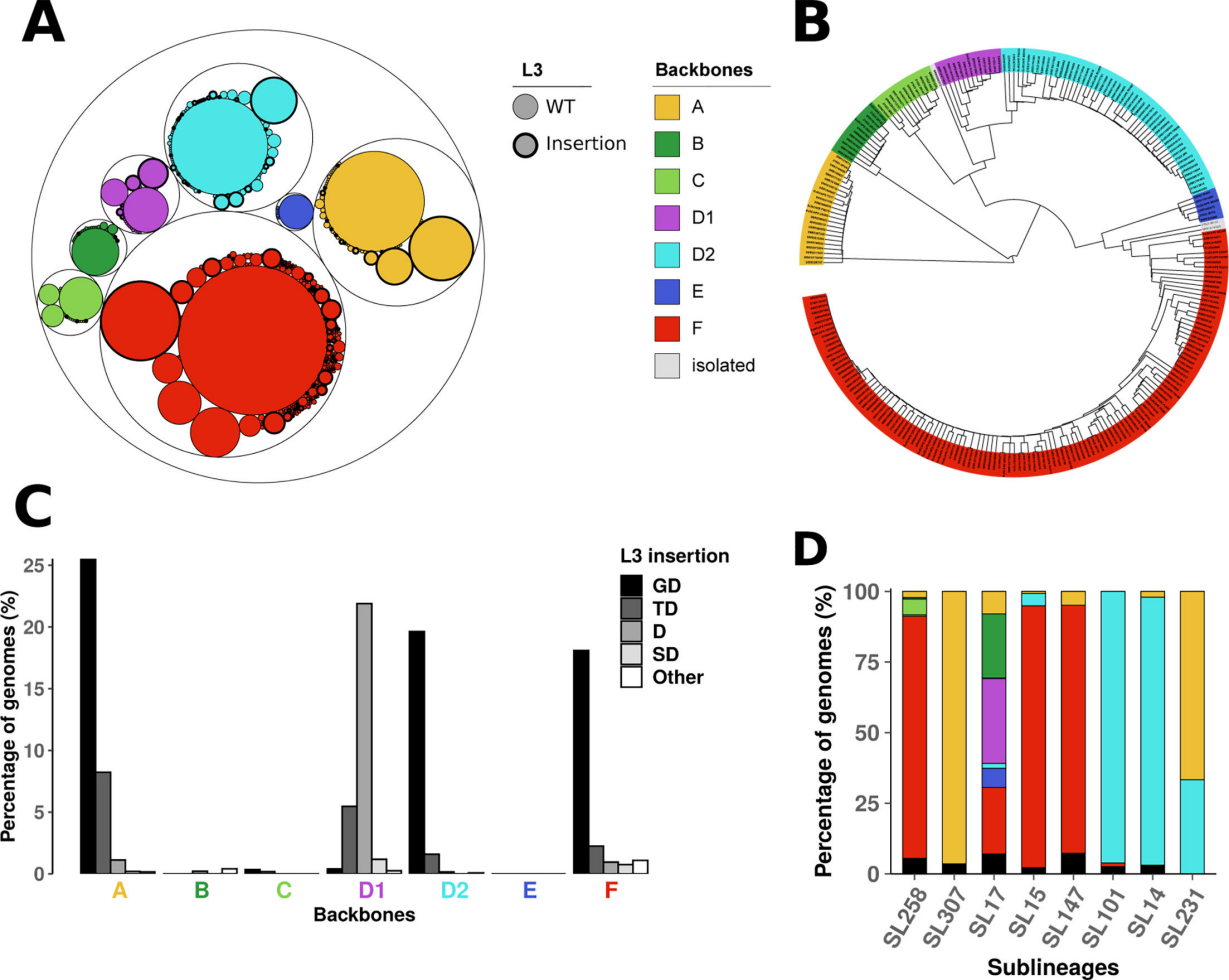
OmpK36 variants and backbones. (**A**) Frequency of OmpK36 variants. Each circle represents a variant and is proportional to its occurrence among the 15,451 genomes from Pathogenwatch database where intact OmpK36 was found. Circles are colored based on backbones. Circles corresponding to variants with L3 insertions are highlighted with thick borders. All variants and their occurrences are available in Table S1. (**B**) UPGMA dendrogram built from protein identity comparison between the 250 concatenated loop variants identified. The backbones correspond to clusters of similar concatenated loop variants and are highlighted in color. (**C**) Frequency of L3 insertions among each backbone. The different L3 insertions are colored in a gray gradient according to the figure key and backbone letters are highlighted in color as for panel A and B. (**D**) Distribution of OmpK36 backbones among the main sublineages (SL) encompassing high-risk clones in the LIN 5 data set: SL14 (*n* = 98), SL15 (*n* = 138), SL17 (*n* = 340), SL101 (*n* = 77), SL147 (*n* = 82), SL231 (*n* = 6), SL258 (*n* = 182), SL307 (*n* = 56). SL are ordered from left to right according to their decreasing prevalence among the 16,086 genomes from Pathogenwatch. Truncated OmpK36 are represented in black. For the sake of readability, variants belonging to backbones Is-1, Is-2, and Is-3 (isolated, *n* = 10) are not represented on panels A and C. These rare backbones were never observed in the high-risk clones presented on panel D. Panel A has been generated with ggraph (18).

Based on 16,086 *Klebsiella pneumoniae* subsp. *pneumoniae* genomes from Pathogenwatch (17), we identified 385 unique, intact OmpK36 protein sequences in 15,451 genomes (Table S1). The remaining 635 genomes carried truncated OmpK36. Three variants were overrepresented, accounting for more than 52% of the genomes belonging to 358 different sublineages: variants 21523_2#103 (*n* = 4,311), 21523_2#104 (*n* = 1,935), and 21523_2#10 (*n* = 1,804) (Fig. 1A; Table S1). We concatenated sequences of the extracellular loops (L1–L2, L4–L8) and the constriction loop L3 (5), identifying 250 distinct concatenated loop variants (Fig. S1). These regions, chosen for their higher variability compared to other parts of the protein (Fig. S2), comprise 38.4% to 42.9% of the full OmpK36 sequence. Then, we clustered these concatenated loops based on percent identity and identified six clusters, along with three rare, isolated sequences (Is-1, Is-2, and Is-3). These represent highly uncommon sequences, characterized by unique patterns in loop L5 for backbones Is-1 and Is-2, and in loop L8 for Is-3 (Fig. S3). We further subdivided one of the clusters (cluster D) to account for variability in loop L8, ultimately defining backbones A, B, C, D1, D2, E, and F (Fig. 1B). OmpK36 variants sharing the same backbone were more similar than variants from different backbones (Fig. S4). Three backbones were dominant in the data set: backbone F (*n* = 7,709, 49.9%), A (*n* = 3,111, 20.1%), and D2 (*n* = 2,583, 16.7%) (Fig. 1A; Table S1). Amino acid insertions in L3 varied among backbones (Fig. 1C), with insertions of “GD” frequent in backbones A (*n* = 794, 25.5%), D2 (*n* = 507, 19.6%), and F (*n* = 1,395, 18.1%), and insertions of “D” enriched in backbone D1 (*n* = 168, 21.9%). Rare backbones B (*n* = 480, 3.1%), C (*n* = 554, 3.6%), and E (*n* = 237, 1.5%) showed only 3, 3, and 0 cases of insertion in L3, respectively.

Simultaneous truncation of the other major porin coding gene, *ompK35*, was observed associated with the seven backbones although with substantial variation in frequency: 44.33% in backbone A, 7.08% in B, 8.30% in C, 32.99% in D1, 21.37% in D2, 19.83% in E, and 62.90% in F. These truncations were strongly associated with the presence of L3 insertions as it was observed in 32.86% of isolates with wild-type L3, compared to 89.71% of those harboring L3 insertions in Pathogenwatch.

### Associations between backbones and resistance classes primarily reflect their prevalence among high-risk clones

To examine the distribution of OmpK36 backbones among high-risk clones, we filtered closely related genomes by clustering the Pathogenwatch database using core genome Multi Locus Sequence Typing (cgMLST) LIN (19) level 5 (≤ 10 differences in cgMLST out of 629 core genes), as recently proposed by Hennart et al. (20). All clusters but one expressed a single OmpK36 backbone, and we selected representative genomes of each cluster to form a data set of 4,228 genomes (Table S1) carrying backbones A (*n* = 920, 21.8%), B (*n* = 226, 5.3%), C (*n* = 254, 6.0%), D1 (*n* = 257, 6.1%), D2 (*n* = 1,051, 24.9%), E (*n* = 127, 3.0%), F (*n* = 1,218, 28.8%), Is-2 (*n* = 8, 0.2%) or truncated (*n* = 167, 3.9%) (Table S2). Their distribution among eight sublineages (SL) which encompass the main STs considered “high-risk clones” (12) (Table S3) showed that each sublineage is associated with a predominant backbone, except SL17 (Fig. 1D). In fact, SL17 contains several major Sequence Types (STs) associated with specific backbones: ST16, ST17, and ST20 associated with backbones D1, B, and F, respectively. We found high frequencies of “D” insertion in L3 of backbone D1 and “TD” insertion in backbone A (Fig. 1C), mainly explained by clonal expansions in ST16 and ST231, respectively, as reported by David et al. (12). Among the 10,331 genomes belonging to high-risk clones in Pathogenwatch, 64.2% (*n* = 6,294) carried backbone F.

**Fig 2:**
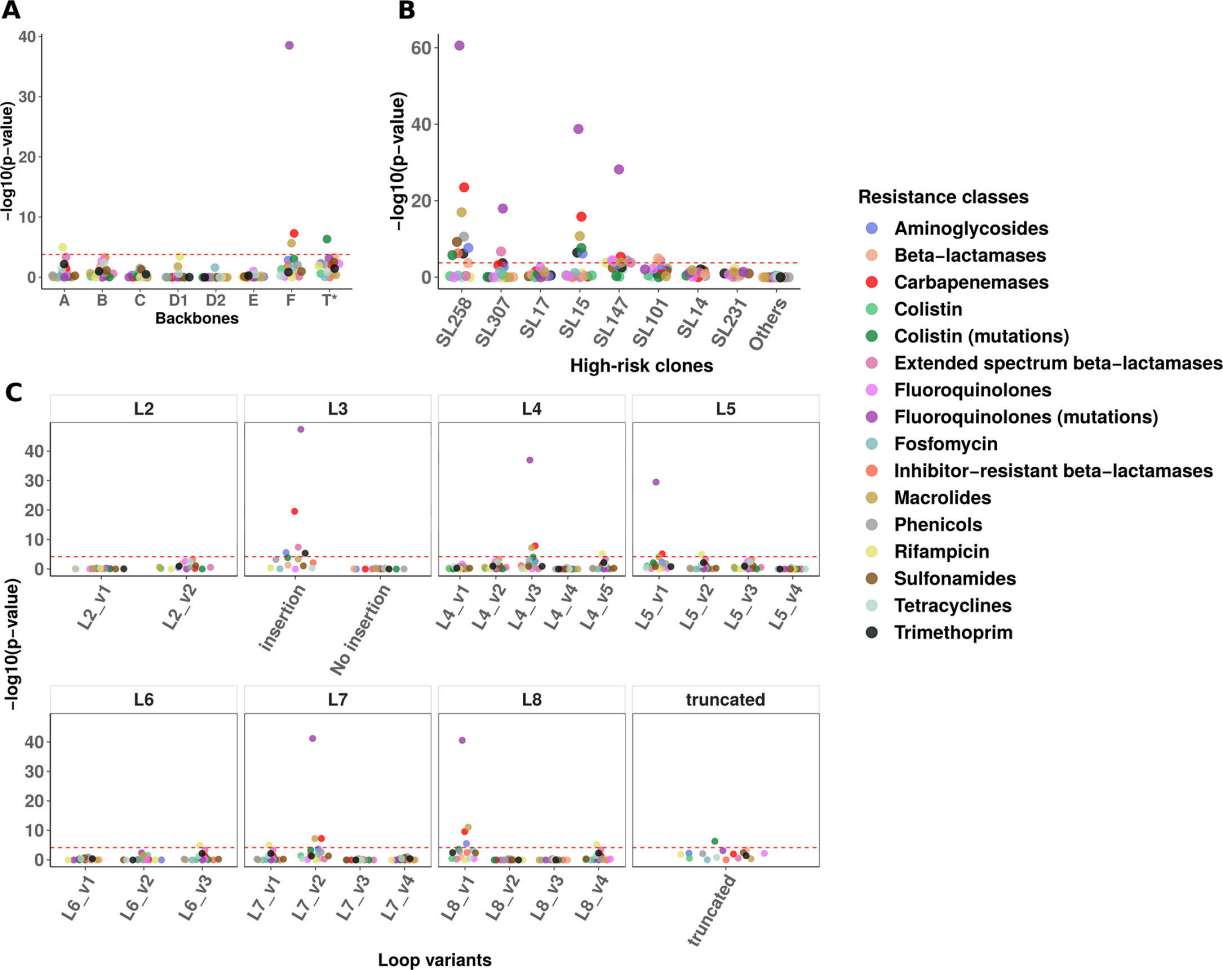
Association analysis of resistance classes with backbones, high-risk clones, and individual loop variants. (**A**) Association analysis between antibiotic resistance classes and OmpK36 backbones. *T = truncated OmpK36. (**B**) Association analysis between resistance classes and the main high-risk clones. The "Others" group includes genomes that do not belong to these clones. (**C**) Association analysis between resistance classes and the main individual loop variants (i.e., prevalence >1%). The amino acid sequence and prevalence of these individual loop variants are available in Table S4. For each panel, the negative logarithm of the association *P*-value for each resistance class is indicated by colored dots. The dashed red line represents the significance threshold after correction of multiple tests by the Bonferroni method.

Since OmpK36 plays a major role in antibiotic permeability, we analyzed associations between resistance determinants, backbones, high-risk clones, and individual loop variants among the LIN 5 data set. We screened each genome for resistance genes and mutations using Kleborate (21), and we used the presence/absence patterns of the corresponding resistance classes to identify associations using coinfinder (22). We found only a few significant associations with backbones (Fig. 2A). Backbone F correlated with fluoroquinolone resistance mutations, carbapenemases, and macrolide resistance genes. Backbone A was linked to rifampicin resistance and truncated OmpK36 to colistin resistance mutations. These associations were largely driven by high-risk clones, particularly SL258 (ST11, ST258, ST512), SL15 (ST15), and SL147 (ST147) for backbone F (Fig. 1D and 2B). Rifampicin resistance was not associated with high-risk clones, but primarily with backbone A in ST873 strains (*n* = 68/81), representing 13.0% of genomes carrying rifampicin resistance genes.

Further analysis at the individual loop variant level identified significant associations with resistance classes (Table S4). Fluoroquinolone resistance mutations, carbapenemases, and macrolide resistance were primarily associated with specific individual loop variants (L3 insertion, L4_v3/v5, L5_v1/v2, L6_v3, L7_v1/v2, L8_v1/v4), mainly found in backbones A and F (Fig. 2C; Fig. S5).

### OmpK36 backbones are scattered throughout the phylogeny of the species, as a result of multiple recombination events

**Fig 3:**
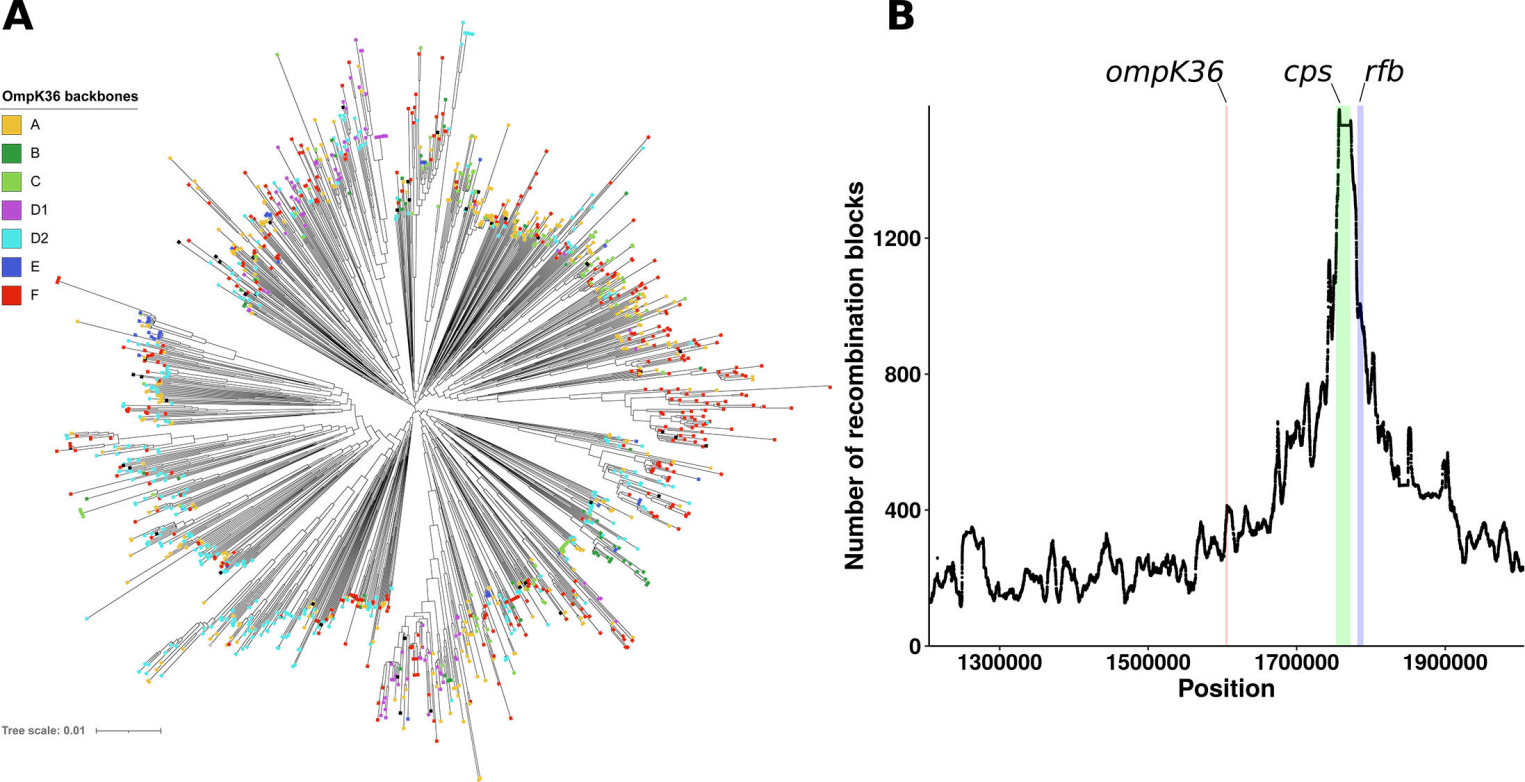
Distribution of Omp36 backbones at the species level. (**A**) Core-genome-based phylogenetic tree of the 1,471 genomes from Pathogenwatch LIN 4 data set. Squares at the branch tips are colored according to OmpK36 backbones. For the sake of readability, seven very long branches were removed (genomes EuSCAPE ES208, ERR3416221, ERR2797011, ERR3416391, DRR124985, ERR2397577, SRR3219817). The tree is mid-point rooted. (**B**) Predicted recombination events in the region of the *rfb* operon, *cps* operon, and *ompK36*. Coordinates are shown on the *x* axis and refer to the reverse positions in bp of *K. pneumoniae* HS11286 reference genome (RefSeq accession number: NC_016845.1). The number of recombination blocks identified using Gubbins is shown on the *y* axis. The full results of the genome-wide recombination analysis are available in Fig. S7.

To assess the phylogenetic distribution of OmpK36 backbones, we analyzed a subset of 1,471 *K. pneumoniae* genomes (Table S5). These genomes were obtained by clustering of Pathogenwatch sequences based on LIN code level 4 (≤ 43 differences in cgMLST), which roughly corresponds to clonal groups (20). They carried OmpK36 backbones A (*n* = 347, 23.6%), B (*n* = 61, 4.1%), C (*n* = 118, 8.0%), D1 (*n* = 93, 6.3%), D2 (*n* = 369, 25.1%), E (*n* = 46, 3.1%), F (*n* = 389, 26.4%) or truncated OmpK36 (*n* = 47, 3.2%). Core-genome-based phylogeny revealed that backbones were scattered throughout the species tree (Fig. 3A; Fig. S6), suggesting multiple recombination events. Recombination analysis using Gubbins (23) confirmed frequent events in the *ompK36* region (Fig. 3B; Fig. S7), which is located near a major recombination hotspot including the capsule biosynthesis (*cps*) and O antigen biosynthesis (*rfb*) operons (24–26).

### *In silico* prediction of pore radius revealed small but significant differences between OmpK36 backbones with wild-type loop 3

**Fig 4:**
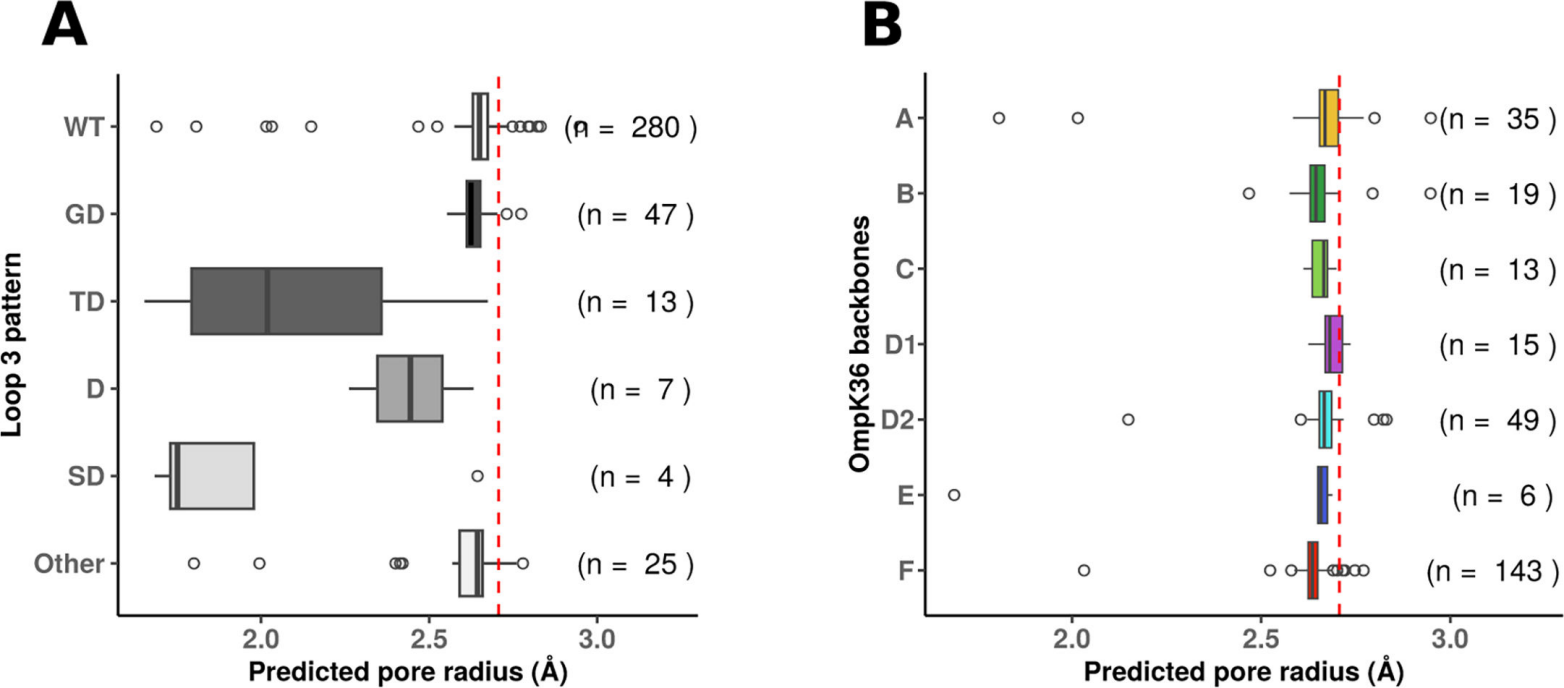
Predicted radius of OmpK36 pores. (**A**) Distribution of pore radius according to insertions in loop L3. The corresponding statistics are available in Table S7. (**B**) Distribution of pore radius according to OmpK36 backbones. The corresponding statistics are available in Table S8. In B, only variants with a wild-type constriction pattern (i.e., with the PEFGGDTYGS pattern) are considered. The red dotted line represents the radius predicted from the OmpK36 reference sequence (PDB ID: 5O79, 2.71 Å), and the number of porin variants is shown on the right. For the sake of readability, variants belonging to backbones Is-1, Is-2, and Is-3 are not represented.

We then predicted 3D structures with Alphafold2 (27) and minimal radii of the pores with HOLE (28). All 3D predictions were of high confidence, with a mean predicted local distance difference (pLDDT) greater than 92/100 in all but one structure (SRR7828806: pLDDT = 87.58). We were able to predict minimal radii for all 385 OmpK36 variants except six that were discarded (Table S6). In agreement with previous reports (10, 12), “GD” (median radius = 2.64 Å, IQR = 0.04), “TD” (median radius = 2.02 Å, IQR = 0.57), “D” (median radius = 2.44 Å, IQR = 0.20), and “SD” (median radius = 1.75 Å, IQR = 0.25) L3 insertions significantly reduced the pore radius compared to the wild-type L3 constriction region (median radius = 2.65 Å, IQR = 0.04) (Fig. 4A; Table S7). The insertions “TD” and “D” were also associated with smaller radius than insertions “GD” and “Other.” Among variants with wild-type constriction region (the PEFGGDTYGS pattern in L3), backbone F had a smaller radius than backbones A, D1 and D2, while backbone B showed a smaller radius than backbone D1 (Fig. 4B; Table S8).

### No major differences in terms of fitness and antibiotic resistance depending on backbones

To assess the functional impact of OmpK36 variation*,* we constructed mutants derived from the strain *K. pneumoniae* CIP110798 (OmpK36 backbone D2). One mutant was deleted for *ompK35* and *ompK36* (CIPΔ*ompK35*Δ*ompK36*). Five additional mutants were constructed by deleting *ompK35* and replacing *ompK36* in strain CIP110798 with the most prevalent *ompK36* variants corresponding to backbones A (21523_2#104, *n* = 1,935), B (21523_2#138, *n* = 429), D1 (21523_2#16, *n* = 387), D2 (21523_2#10, *n* = 1,804), and F (21523_2#103, *n* = 4,311), respectively. Due to the absence of strains carrying the minor backbone C and E in our collection, these backbones were not included in further experimental studies. We examined the effect of *ompK36* variants in a strain deleted for *ompK35*, as this gene is frequently inactivated in *Klebsiella pneumoniae,* such as ST258 strains (9). Moreover, deleting *ompK35* allows us to specifically assess the impact of OmpK36 protein variants on permeability.

**Fig 5:**
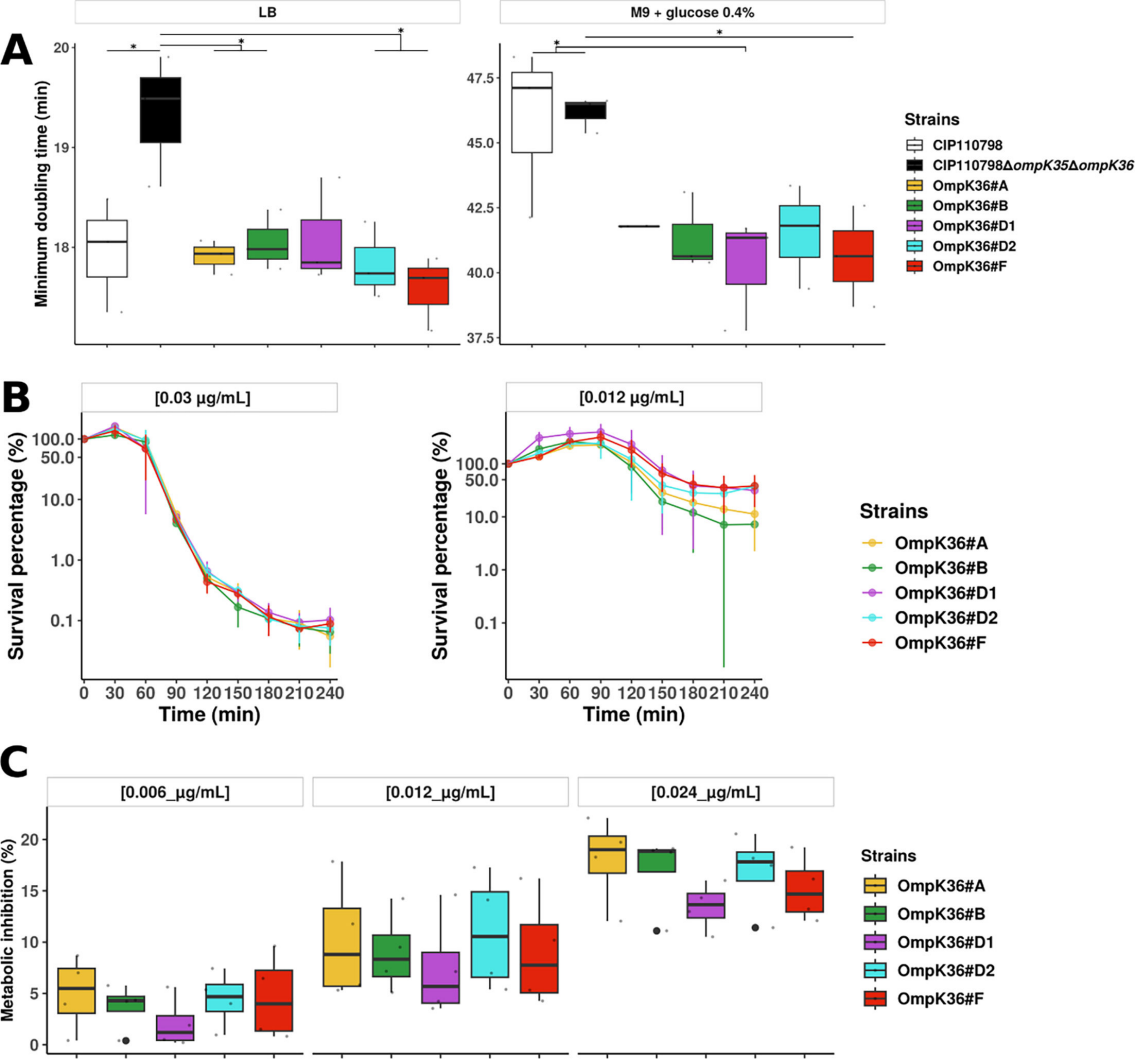
Phenotypic comparisons of wild-type and *ompK36*-variant strains. (**A**) Minimal doubling time (minutes) in rich (LB) and minimal media (M9 + glucose 0.4%) of the parental strain (OmpK36 backbone D2), the double deleted strain, and the ∆*ompK35* variants with OmpK36 backbone A, B, D1, D2, and F. (**B**) Time-kill curves for OmpK36 mutants in the presence of ertapenem 0.03 µg/mL or 0.012 µg/mL over 4 h. (**C**) Metabolic inhibition of OmpK36 mutants measured in the presence of resazurin and ertapenem at 0.006, 0.012, and 0.024 µg/mL. The percentage of metabolic inhibition was calculated as the difference in relative fluorescence units (*λ*_excitation_ = 530 nm and *λ*_emission_ = 590 nm) with and without ertapenem at the first plateau point of the growth curve (29).

Minimum doubling time computed from growth curves in rich (LB Miller) and minimal (M9 + glucose 0.4%) media showed no major fitness differences between *ompk36* variants. However, the double deleted strain exhibited an increased doubling time in rich media (Fig. 5A) compared to the WT and all mutants except OmpK36#D1 (*P* = 0.057). In minimal medium, the WT and double-deleted strains grew more slowly than the mutant strains. However, due to experimental variations, the differences were only significant for the WT strain compared with OmpK36#D1 (*P* = 0.040) and for the double-deleted mutant compared with OmpK36#D2 (*P* = 0.028) and OmpK36#F (*P* = 0.042).

We then measured the minimum inhibitory concentrations (MICs) of seven antibiotics, including four antibiotics generally affected by reduced permeability (cefotaxime, nalidixic acid, cefoxitin, and temocillin). We did not observe significant differences between the five *ompk36* variants (Table 1). The CIPΔ*ompK35*Δ*ompK36* mutant displayed a MIC increase for all antibiotics except for imipenem, in line with previous studies on porin mutants lacking carbapenemase (11). Compared to the WT, *ompk36* variants showed increased MICs for cefotaxime, nalidixic acid, cefoxitin, and temocillin, as well as a twofold increase for ertapenem (0.024 vs 0.012 µg/mL), but only using microdilution. Microdilution resulted in higher MICs of ertapenem compared with E-test, which may be due to the variation in growth depending on the solid/liquid media.

**TABLE 1:** Minimum inhibitory concentrations of antibiotics for the different strains

Strain	Genotype	Predicted radius OmpK36 (Å)	E-test MIC (µg/mL)							Microdilution MIC (µg/mL)
			ETP^*a*^	IMP	MP	CTX	NAL	FOX	TEM	ETP
CIP110798	WT (OmpK36 backbone D2)	2.665	0.003	0.19	0.032	0.004	2	0.75	1	0.012
CIPΔ*ompK35*Δ*ompK36*	Δ*ompK35*Δ*ompK36*	/	0.25	0.19	0.125	0.25	3	48	8	0.5
Δ*ompK35* OmpK36#A	Δ*ompK35*Δ*ompK36::ompK36-backboneA*	2.723	0.003	0.19	0.032	0.047	3	4	6	0.024
Δ*ompK35* OmpK36#B	Δ*ompK35*Δ*ompK36*::*ompK36*-backboneB	2.645	0.003	0.19	0.032	0.047	4	4	6	0.024
Δ*ompK35* OmpK36#D1	Δ*ompK35*Δ*ompK36*::*ompK36*-backboneD1	2.669	0.003	0.19	0.032	0.047	4	4	6	0.024
Δ*ompK35* OmpK36#D2	Δ*ompK35*Δ*ompK36*::*ompK36*-backboneD2	2.665	0.003	0.19	0.032	0.047	3	4	6	0.024
Δ*ompK35* OmpK36#F	Δ*ompK35*Δ*ompK36*::*ompK36*-backboneF	2.647	0.003	0.19	0.032	0.064	4	4	6	0.024

^*a*^ ETP, ertapenem; IMP, imipenem; MP, meropenem; CTX, cefotaxime; NAL, nalidixic acid; FOX, cefoxitin; TEM,temocillin.

We also determined the MICs to four antibiotics (ertapenem, imipenem, meropenem, and cefotaxime) of the same strains expressing the carbapenemase coding gene *bla*_KPC-2_, under the control of a p_BAD_ promoter. As expected, the presence of the carbapenemase markedly increased antibiotic MICs (Table S9), particularly for the double deleted strains and the mutants. However, no difference was also observed between the different *ompK36* variants tested.

Next, we focused on the OmpK36#A to F variants, which all showed identical MICs of ertapenem in microdilution. First, we performed time-kill assays for 4 h, looking for early differences that might result from differences in permeability. Regardless of the ertapenem concentration tested (0.03 or 0.012 µg/mL), no differences were observed between variants (Fig. 5B). Second, to assess antibiotic permeation (29), we performed an antibiotic uptake assay using resazurin in the presence/absence of ertapenem. This assay revealed no significant differences in metabolic inhibition across the range of ertapenem concentration tested (0.006, 0.012, or 0.024 µg/mL) (Fig. 5C).

**Fig 6:**
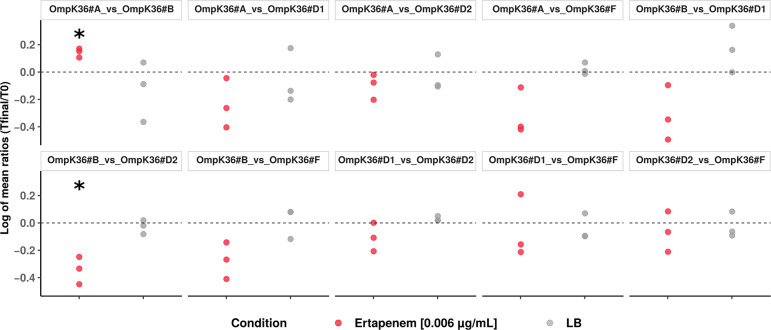
Competition assay between the mutant strains in LB Miller without antibiotic and MHB with subinhibitory concentration of ertapenem (0.006 µg/mL). Gray and red points indicate the log of mean ratio of Sanger peak amplitude at the end of experiment over ratio of Sanger peak amplitude at T0 for a given experiment in LB Miller and MHB + ertapenem, respectively. Statistically significant differences were searched for a given pair of mutants in a given condition (LB or MHB + ertapenem) by comparing the log of mean ratio to 0 (i.e., no difference of fitness) using a one-sample *t*-test. Significant results are highlighted by asterisks.

Finally, in order to increase the sensitivity of the mutant comparison, we performed competition assays over 53 generations (7 passages) with ertapenem at sub-inhibitory concentration (0.006 µg/mL) and without. In agreement with the growth curve data (Fig. 5A), we did not observe fitness differences in LB Miller without ertapenem (Fig. 6). However, in MH broth (MHB) + ertapenem, OmpK36#B was outcompeted by OmpK36#A (*P* = 0.018) and OmpK36#D2 (*P* = 0.027) and seemed less fit than OmpK36#F but with a weaker signal (*P* = 0.072) (Fig. 6). Note that we observed substantial inter-experimental variation which might have obscured existing differences between other variants.

### OmpK36 backbones exhibit differences in phage susceptibility and mutations in loop 4 may lead to phage resistance

Phages have been largely documented as strong drivers of diversification on outer membrane proteins, particularly porins, which some phages use as receptors for attachment to their hosts (30). In this context, we hypothesized that some phages may be specific to particular backbone. First, we infected an *E. coli* MG1655 strain and a double mutant Δ*ompC*Δ*ompF* (Fig. S8A) with two well-documented *E. coli* phages that used OmpC as their receptors: the T4 phage, and Bas44 phage from the Basel phage collection (Genbank accession number: MZ501046.1) (31). T4 infected *E. coli* Δ*ompC*Δ*ompF,* consistent with previous observations that T4 can also recognize the LPS core (32) (Fig. S8B). Bas44, however, showed clear porin-dependent infection. Therefore, we used Bas44 to infect an *E. coli* MG1655 Δ*ompC*Δ*ompF* strain complemented with the five *ompK36* variants expressed from pACY plasmid derivatives (Fig. S8A). We observed lysis plaques on *E. coli* Δ*ompC*Δ*ompF* carrying OmpK36 backbones D1 and D2, but not A, B, and F (Fig. S8C and D). Whole-genome sequencing of an isolated phage plaque revealed how mutations in phage genes were selected against different OmpK36 variants: a substitution in the receptor-binding protein (Bas44_0084), A978D for both variants *ompK36*#D1 and *ompK36*#D2 (phage mutant Bas44_ompK36#D1), and a substitution, D100G, in a putative RNAseH gene (Bas44_0080) for variant *ompK36*#D2 only (phage mutant Bas44_ompK36#D2).

**Fig 7:**
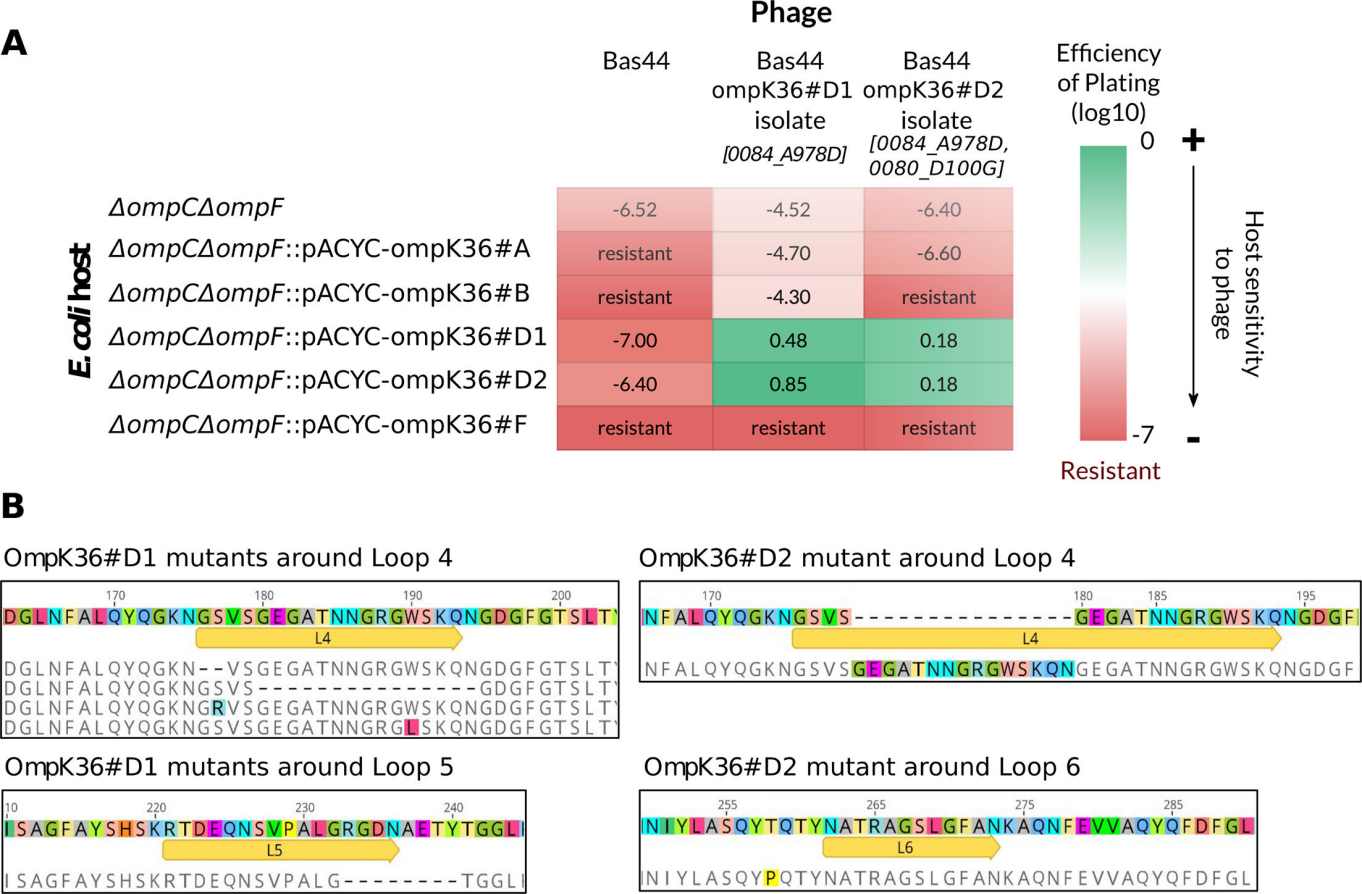
Isolation of *ompK36* mutants resulting from phage selection. (**A**) *E. coli* mutant strains susceptibility toward Bas44 phages before and after amplification on susceptible strains. The efficiency of plating is represented by a color gradient, ranging from green for susceptibility to red for resistance. (**B**) Protein sequence variants of porin mutants resulting from phage selective pressure obtained for OmpK36#D1 and OmpK36#D2. A segment duplication, deletions, and non-synonymous substitutions happened in Loop 4, a deletion happened in Loop 5, and a non-synonymous substitution happened close to Loop 6. These mutations are highlighted in color or by dashes.

Infections of *E. coli* MG1655 harboring the five *ompk36* variants using these two phage mutants resulted in strikingly different susceptibility patterns (Fig. 7A). OmpK36 variants D1 and D2 were both infected by phages Bas44_ompK36#D1 and Bas44_ompK36#D2, while showing poor susceptibility to the Bas44 wild-type phage. Interestingly, backbones D1 and D2 have similar loop sequences, differing mainly in loop 8 (Fig. S3).

Next, we assessed the impact of phage selective pressure on OmpK36 by infecting *E. coli* MG1655 Δ*ompC*Δ*ompF* complemented with *ompK36*#D1 or *ompK36*#D2 variants using their respective phages, Bas44_ompK36#D1 and Bas44_ompK36#D2. We amplified and sequenced *ompK36* in 16 resistant colonies isolated for each *ompK36* variant. Most resistant colonies (9/16 and 14/16, respectively) were mucoid and had no mutation in *ompK36*, a known mechanism of resistance in *E. coli* toward a broad range of phages (33, 34). Among non-mucoid clones, most mutations were located in loop 4 (*n* = 7/9), with only one mutation found in loops 5 and 6, respectively (Fig. 7B). Two mutations in loop 4 Δ(181–195) and S177R were found twice.

Finally, we attempted to test phage pressure directly in *K. pneumoniae* by infecting CIP110798 and the mutant strains expressing the five *ompK36* variants using both Bas44, Bas44_ompK36#D1 and Bas44_ompK36#D2. However, none of these phages was able to propagate in the *K. pneumoniae* strains tested (Fig. S9).

## DISCUSSION

Outer membrane porins (OMPs) are the main gateway for small hydrophilic molecules, nutrients, and toxic compounds, into the periplasm of Gram-negative bacteria. They are also receptors of phages and bacteriocins, making them subject to diverse and sometimes conflicting selective pressures. In *K. pneumoniae*, OmpK36, the ortholog of *E. coli* OmpC porin, plays a significant role in reduced permeability to β-lactams, particularly through amino acid insertions in the L3 constriction loop (10, 11, 15, 16). These insertions contribute to β-lactam resistance, further enhanced by carbapenemase. This combination is prevalent in “high-risk clones,” which are major drivers of antibiotic resistance (3). Similar patterns have been described in *E. coli*, where recombination involving a specific *ompC* allele, particularly from the multiresistant ST38 clone, often precede the acquisition of carbapenemase coding gene (35). However, aside from L3 insertions, the diversity of OmpK36 and its impact on antibiotic resistance and phage susceptibility remains poorly understood. To investigate this, we analyzed over 16,000 genomes of *K. pneumoniae* subsp. *pneumoniae*, revealing significant diversity in OmpK36, particularly in the extracellular loops. We identified seven main backbones of OmpK36, which are non-randomly associated with the so called high-risk clones. Phylogenetic analysis indicates that recombination also plays a key role in the spread of these variants in this species. Additionally, some of these backbones showed reduced pore size based on their predicted 3D structures.

Despite this genotypic diversity, we found no major differences in antibiotic resistance or fitness between mutants carrying five of the seven identified backbones. We used both static (E-test, broth microdilution) and more dynamic (resazurin-based assay, competitions with short-term evolution) approaches to evaluate resistance, as well as growth assays under varying osmolarity conditions. No major variability was observed among isogenic strains, except backbone B, which showed increased susceptibility to ertapenem when competing against backbones A, D2 and a trend for backbone F. Backbone B was rarely observed in both the population and high-risk clones. It is, therefore, tempting to hypothesize that increased permeability to antibiotics could explain its lower prevalence. It is also interesting to note that backbone B, like backbones C and E, is one in which L3 insertions are very rare. Increased permeability resulting in a reduced ability to survive low concentrations of antibiotics could have limited the probability of selecting L3 insertions. Alternatively, structural constraints on these backbones may also limit the acquisition of such insertions. The *in vitro* evolution of clones carrying these backbones in the presence of antibiotics would probably make it possible to test this last hypothesis. In any case, it seems that antibiotic pressure is not responsible for all the diversity observed in OmpK36 and that it essentially selects for L3 insertions.

Phage susceptibility, another selective pressure encountered by bacteria, revealed greater differences between OmpK36 backbones. Our analysis confirmed that OmpC-like porins such as OmpK36 are a major entry route for some phages (36, 37), including Bas44, which showed a backbone-dependent host range. Plaque lysis analysis indicated rapid evolution toward a greater infectivity against the initial backbone-bearing strain and the location of phage mutations suggest a direct involvement of the tail fiber in this adaptation to the host. As bacterial-phage interactions are often described as an evolutionary arms race (38), we also screened for resistant bacterial mutants. Most displayed non-specific resistance via increased mucoidy, a known defense mechanism (33). However, several mutants showed convergent evolution with mutations in extracellular loops, particularly in loop 4 (found in 7/9 resistant clones), as well as in loops 5 and 6. These findings align with prior studies showing that loops 4, 6, and 8 in *K. pneumoniae* OmpK36 (39) and loops 1, 4, and 5 in *E. coli* OmpC play a crucial role in phage adsorption (40). Overall, this suggests that extracellular loop variations, especially in loop 4, are key determinants of phage-host interactions and may be subject to strong selective pressure.

Our study has limitations. First, part of our work relies on *in silico* predictions, which may result in misprediction of porin size, and we did not account for the electric field within the pore, an important property of OMPs (41), nor for the dynamics of the internal loop (42). Nonetheless, our analysis confirmed the expected reduction in pore size when insertions in L3 are present. Second, while we tested the MICs of several antibiotics, we performed an in-depth analysis of susceptibility to ertapenem only. We chose to focus specifically on this carbapenem, as identifying differences in susceptibility to a last-line antibiotic may have significant clinical implications. Nevertheless, a more detailed analysis of susceptibility to other antibiotics would be highly valuable and warrants further investigation in future studies. Third, we only tested two phages, while a more comprehensive analysis of phage-host interactions (43, 44) could provide a deeper understanding of phage-driven selection. But such an extensive analysis of phage host range was beyond the scope of this study. In the future, it will be also valuable to consider the selective pressure that might be imposed by the host immune system, which could also play a pivotal role through the complement for example (45, 46). Fourth, Bas44 and its derivatives could not infect *K. pneumoniae* expressing different *ompK36* variants. However, although less direct, phage pressure on *E. coli*, assumed to rely on the OmpK36 attachment, can simulate the types of structural changes these proteins could undergo to escape phage attachment. Finally, we were unable to test two of the less frequent backbones (C and E) because no corresponding strain was available in our collection for mutant construction. They would merit further study.

In conclusion, we have highlighted the substantial diversity of the porin OmpK36. Different selective pressures seem to act on different parts of the protein and could partly explain this diversity. Antibiotics mainly select for L3 insertions, which are linked to resistance (10–12, 15, 16), but the diversity outside this region does not correlate with major differences in antibiotic susceptibility. Phages, on the other hand, exhibit strong specificity for certain backbones and individual loop variants, and bacterial resistance to phages can evolve rapidly via mutations in extracellular loops. Overall, our study highlights the importance of considering the diverse selective pressures faced by porins to better understand how different variants are selected, and the role they play in bacterial pathogenesis and evolution.

## MATERIALS AND METHODS

### OmpK36 data set

*K. pneumoniae* genomes were downloaded from Pathogenwatch (17) (23 November 2021) with cgMLST and Kleborate v2.0.4 data (21). Coding sequences were predicted using Prodigal v2.6.3 (47) and aligned against the OmpK36 reference sequence (Uniprot accession: Q48473) using diamond v2.0.4 (48) (⩾ 80% protein identity and ⩾ 90% alignment length). OmpK36 sequences were extracted with samtools v1.9 (49), aligned with MAFFT v7.407 (50), and then filtered in jalview v2.11.1.4 (51). Gene synteny was verified by detecting at least one of the expected upstream (*rcsC*, *rscB*, *rcsD*) or downstream (*apbE*, *alkB*, *ada*, *yojI*) genes.

### Clustering of OmpK36 porins

OmpK36 variants were classified into “backbones” based on extracellular loops (L1, L2, L4–L8) and constriction loop L3 amino acid sequences. For each variant, loop sequences, as defined by Alberti et al. (5), were extracted and concatenated. Then, sequences were aligned with clustalo v1.2.4 (52) and clustered based on pairwise percent identities using hclust from R package “stats,” with the method “centroid” and a threshold of 95%. A UPGMA tree was also built from the protein identities after alignment in jalview with MuscleWS v3.8.31 (53) and visualized with Itol (54). For each backbone, consensus sequences were generated with “cons” from EMBOSS v6.5.7.0 (55) and visualized in MView (56).

### cgMLST-based Pathogenwatch clustering

Genome redundancy was reduced based on Life Identification Numbers (LIN) codes from Pathogenwatch (17, 20). A first clustering at LIN level 5 (“LIN five data set”) removed very closely related genomes with ≤10 cgMLST allele mismatches. From this data set, we analyzed the distribution of backbones among eight sublineages encompassing the ST considered as high-risk clones by David et al. (12): SL14 (ST14), SL15 (ST15), SL17 (ST16), SL101 (ST101), SL147 (ST147), SL231 (ST231), SL258 (ST11, ST258, ST512), SL307 (ST307). SLs are defined based on a 190 allelic mismatch threshold (20). The detailed distribution of STs in the studied SLs is available in Table S3. A second clustering based on LIN level 4 (“LIN 4 data set”) further reduced the data set for phylogenetic analysis (≤43 cgMLST allele mismatches which correspond to clonal groups) (20).

### Analysis of the association of backbones, high-risk clones, and sequences of individual loops with resistance classes

Resistance classes were determined via Kleborate, excluding “Bla_chr,” “SHV_mutations,” and “Omp_mutations.” The classes result from the presence of genes or chromosomal mutations involved in resistance to different antibiotics. Then, we ran Coinfinder v1.2.0 (22) on the LIN 5 data set with the association mode without filtering for high or low abundances to search for associations between (i) backbones and resistance classes and (ii) sublineages and resistance classes. We also searched for association between individual loop variants present in at least 1% of the LIN 5 data set and resistance classes. Briefly, we extracted the amino acid sequences of individual loops L2 and L4 to L8 from genomes and clustered them based on a 100% identity and coverage. L3 variants were classified as “insertion” (GD, TD, D, SD, or other) or “no insertion” (Table S4). Tigecycline (0.1% prevalence) was excluded. Multiple-testing correction using Bonferroni method was applied.

### Core-genome phylogeny and detection of recombinations

We computed a core-genome alignment using snippy-multi (57) from the LIN 4 data set and the *K. pneumoniae* HS11286 (RefSeq accession number: NC_016845.1) as reference. Then, we built a phylogenetic tree using FastTree v2.1.11 (58) with the general time-reversible model and visualized using Itol (54). Recombination was detected with Gubbins v3.2.0 (23) and visualized with Phandango (59).

### OmpK36 porin radius prediction

Three-dimensional structures of OmpK36 variants were predicted using AlphaFold2 (27) (--max_template_date = 2021-12-19, model_preset = monomer, --db_preset = full_dbs). Then, we aligned structures onto OmpK36 reference structure (PDB accession number 5O79) with PyMOL 2.0.1 (60). The mean minimal radius of each variant was estimated from 50 rounds of analysis using HOLE v2.0 (28). For five variants, we obtained only a limited number of HOLE results (6–11 values) and for six variants, we obtained only outliers, which were discarded. Minimal radius was also predicted from the amino acid sequence of the reference structure OmpK36 5O79. We used Kruskall-Wallis test to compare radii across L3 patterns and backbones, followed by Dunn’s tests when significant while adjusting *P*-values for multiple tests (Benjamini-Hochberg method) with the rstatix package (61).

### o*mpK36* mutant construction

We constructed mutant strains deleted for *ompK35* and carrying *ompK36* variants representative of the different backbones. Deletion of *ompK35*, frequent in clinical isolates (*n* = 7,470, 46.4% in the Pathogenwatch database), has been reported to enhance the effect of mutations in *ompK36* (10, 11). Backbones C and E, rare and absent in our strain collection, were not studied. The detailed method is available in supplementary methods. Briefly, we deleted *ompK35* and *ompK36* in *K. pneumoniae* CIP110798 using one-step inactivation (62), yielding CIP110798Δ*ompK35*Δ*ompK36* (designated Δ*ompK35*Δ*ompK36* throughout the manuscript) (Table S10). In parallel, we performed homologous recombination between *ompk36* variants representative of the main OmpK36 porin backbones at the original chromosomal location in the double-deleted mutant yielding mutants CIP110798Δ*ompK35*Δ*ompK36::ompK36*-backbone A, B, D1, D2, and F (designated hereafter OmpK36#A, OmpK36#B, OmpK36#D1, OmpK36#D2, and OmpK36#F). The tested variants correspond to the most prevalent sequence within each backbone in Pathogenwatch database. All mutants were controlled by PCR (Table S10) and sequenced by Illumina 2*150 pb (NEBNext Ultra II FS DNA library prep kit) after DNA extraction using Blood and Tissue DNA easy kit (Qiagen). Genomes were compared with the original strain using Breseq (63) with standard parameters. Sequences are available in the Bioproject PRJEB88512.

In a second step, the carbapenemase coding gene *bla*_KPC-2_ was cloned under the control of a p_BAD_ inducible promotor in a pHV7 plasmid (64). Then, CIP110798, Δ*ompK35*Δ*ompK36* and each mutant were transformed with the resulting plasmid.

### Fitness cost assessment through growth curves in rich and minimal media

Wild-type and mutant strains were grown in LB Miller or in M9 1× minimal media supplemented with MgSO4 1 mM, CaCl2 0.1 mM, and glucose 0.4% (M9G) until exponential phase. From these cultures, we inoculated a microplate containing LB Miller or M9G with 10^6^ CFU/mL and monitored growth by reading of optical density at 600 nm (OD_600_) every 10 min at 37°C with continuous shaking (TECAN Infinite). Strains were analyzed at least in triplicate in three independent experiments. We performed ANOVA on the mean of minimum doubling time for each strain in each experiment. When significant, we performed Tukey’s test while adjusting *P*-values for multiple tests (Benjamini-Hochberg method) with the rstatix package (61).

### Minimum inhibitory concentrations

MICs of ertapenem, imipenem, meropenem, cefotaxime, cefoxitin, temocillin, and nalidixic acid were determined using E-test (Biomerieux) on Mueller-Hinton agar plates incubated at 37°C for 24 h with an inoculum of 10^6^ CFU/mL. We also measured the MIC of ertapenem by microdilution in liquid medium Mueller-Hinton broth (MHB) at 37°C for 24 h.

For mutants complemented with pHV7-*bla*_KPC-2_, strains were grown overnight (ON) in LB + Chloramphenicol 10 mg/L (Cm). Then, ON cultures were diluted 1:100 and *bla*_KPC-2_ expression was induced for 1 h in MH broth + Cm + arabinose 0.4%. MICs of ertapenem, imipenem, meropenem, and cefotaxime were determined on MH agar plates + arabinose 0.4% by using E-test.

### Ertapenem time-kill curves

Mutant strains were grown ON in LB Miller and then diluted 1/1,000 in MHB until exponential phase. Then, we inoculated MHB + ertapenem 0.012 and 0.03 µg/mL at a bacterial concentration of 10^6^ CFU/mL. Cultures were incubated at 37°C with continuous shaking. Viability was assessed by serial dilution and plating on antibiotic-free LB agar plates incubated at 37°C for 24 h. Colonies were counted using Scan 4000 (Interscience), and survival was expressed as percentage of the initial inoculum. Experiments were repeated independently three times.

### Measure of ertapenem permeation through resazurin-based assay

To assess the permeation of ertapenem into mutant strains, we monitored the bacterial metabolic inhibition by measuring resazurin reduction as proposed by Masi et al. (29). Strains were grown ON in LB Miller, diluted 1/200, and incubated until exponential phase. Then, Greiner µClear black microplate (Cellstar, REF:655090) were inoculated with 10^7^ CFU/mL in MHB, 10% (vol/vol) alamarBlue HS Cell Viability Reagent (ThermoFisher Scientific) and ertapenem at various concentrations (0.12, 0.24, or 0.48 µg/mL). Fluorescence (*λ*_excitation_ = 530 nm, *λ*_emission_ = 590 nm) was recorded every 10 min for 5 h under continuous shaking at 37°C (TECAN Infinite). We computed the percentage of metabolic inhibition as the difference between the relative fluorescence units with and without ertapenem at the first plateau point of the curve. Strains were tested at least in duplicate in four independent experiments, and mean metabolic inhibition was compared by ANOVA.

### Competition assays

To compare mutants in competition without adding a selective marker, we took advantage of polymorphisms in *ompK36* sequences as previously proposed (65). Detailed method is available in Supplementary method. Briefly, we mixed calibrated ON cultures of all possible strain pairs (*n* = 10) at a 1:1 ratio. Then, these cultures were diluted 1/200 twice a day in the fresh corresponding medium (LB Miller or MHB + ertapenem) for 3 days. The fourth day we performed subcultures in a 24-well plate (TPP) in the corresponding media until reaching the stationary phase, resulting in a total of approximately 53 generations. For each strain mix, we compared the amplitude of Sanger sequencing peaks at polymorphic site using sangerseqr package (66) following PCR amplification with primers OmpK36_4_F/OmpK36_2_R or OmpK36for/OmpK36_1_R at T0 and Tf in LB Miller and MHB + ertapenem. We computed the mean and standard deviation of the log-transformed average ratio from three independent assays for each pair in each condition and compared it to the value 0 (i.e., no difference between strains) using one sample *t*-test.

### Evolution of *E. coli* phages using OmpK36 porins as receptors and isolation of porin mutants under phage selective pressure

We ran experiments using two well-documented *E. coli* phages which use OmpC as their receptors: the T4 phage and Bas44 phage from Basel collection (31). An *E. coli* K-12 MG1655 Δ*ompC*Δ*ompF* strain was complemented with pACY-derived plasmids carrying *ompK36* representative of backbones A, B, D1, D2, and F (see Supplementary method) and grown ON in LB + Chloramphenicol at 20 mg/L (LB + Cm). Bacterial lawns were prepared by mixing 200 µL of a stationary phase culture with LB + 0.5% agar supplemented with CaCl_2_ 5 mM and MgSO_4_ 5 mM and poured onto LB plates + Cm. Serial dilutions of the phage stocks were spotted on plates and incubated ON at 37°C. Individual plaques (phage variants) were isolated and amplified through liquid infection, using the *E. coli* Δ*ompC*Δ*ompF* strain complemented with the *ompK36* porin gene from which that phage plaque was isolated. We also assayed infection of *K. pneumoniae* CIP110798 and the mutant strains (Δ*ompK35*Δ*ompK36,* OmpK36#A, B, D1, D2, and F) with T4, Bas44, and these phage variants using the same procedure as for *E. coli*. Whole-genome sequencing of phage variants was performed as proposed by Jakociune et al. (67) after Nextera XT DNA library preparation on an Illumina NextSeq500. Mutations were identified using Breseq (v. 0.33.2) (63) against Bas44 genome sequence (NCBI accession number MZ501046).

To search for phage-resistant mutants, an ON culture of each *E. coli* Δ*ompC*Δ*ompF*::pACY-*ompK36* was diluted 1:50 in 1 mL of fresh LB + Cm, mixed with 20 µL of the amplified phage stock (titer ~10^7^ pfu/µL, MOI ~ 1) and grown at 37°C ON with agitation. Lysis of the population by the phage was visible after a few hours. After ON growth, phage-resistant clones were restreaked on LB + Cm agar plates, PCR-screened, and sequenced (Table S10). Porin mutants were then checked by whole-gene and promoter sequencing (Table S10).

## SUPPLEMENTAL MATERIAL

Supplemental figures mbio.01343-25-s0001.pdf Figures S1 to S9.

Supplemental methods mbio.01343-25-s0002.docx *ompK36* mutant construction, construction of pACY plasmids carrying *ompK36* representative, and Sanger sequencing-based competition assays.

Supplemental tables mbio.01343-25-s0003.xlsx Tables S1 to S10.

ASM does not own the copyrights to Supplemental Material that may be linked to, or accessed through, an article. The authors have granted ASM a non-exclusive, world-wide license to publish the Supplemental Material files. Please contact the corresponding author directly for reuse.
