# Supplementary material for: Antibiotics and phages drive region-specific diversity of OmpK36 in Klebsiella pneumoniae
Source: mBio. 2025 Aug 18;16(9):e01343-25. doi: 10.1128/mbio.01343-25 (PMC12421954; doi:10.1128/mbio.01343-25)
Supplement: Supplemental figures — Figures S1 to S9. [file mbio.01343-25-s0001.pdf]

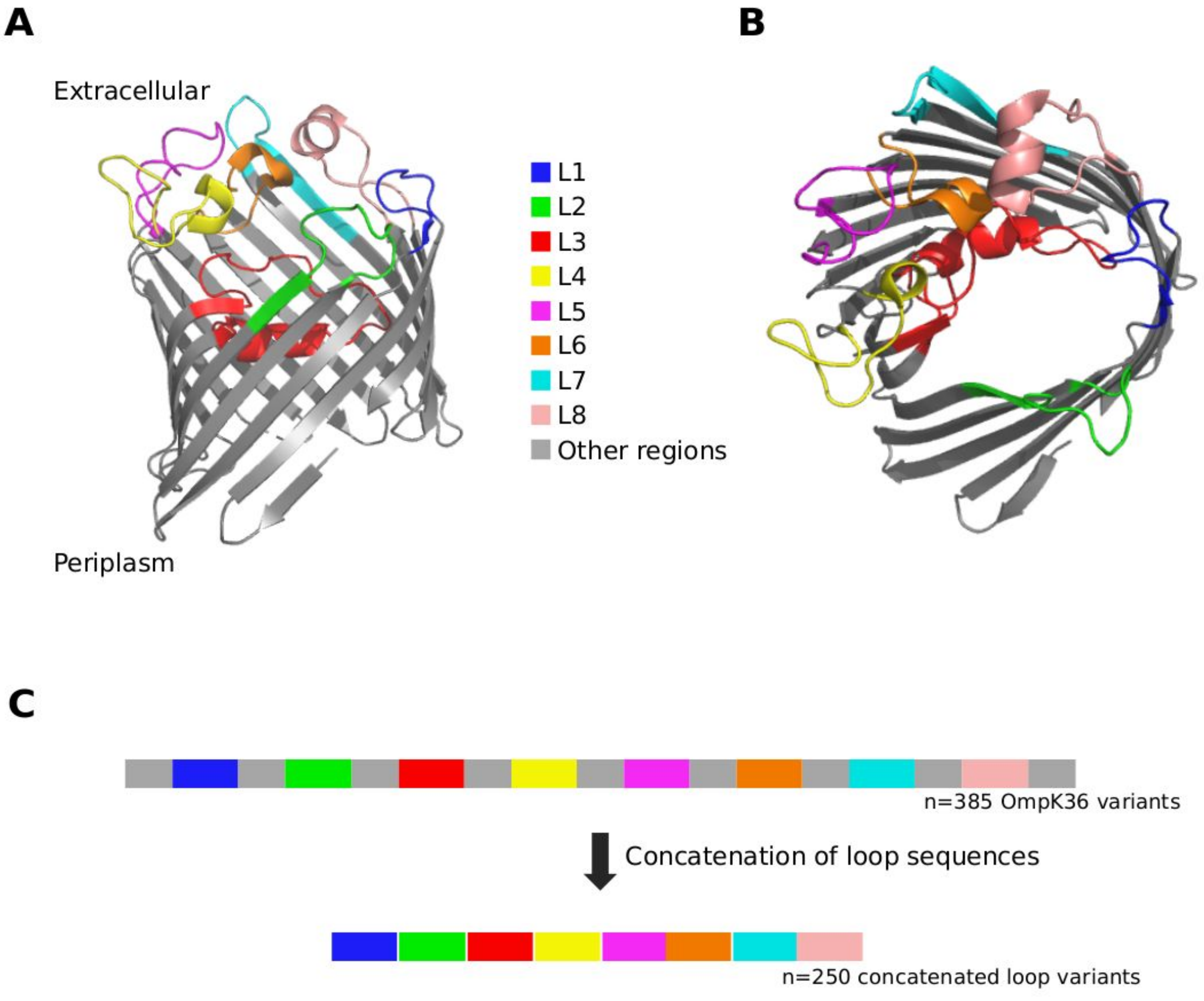

Figure S1. Structure of the OmpK36 porin and its extracellular loops. (A) Side view of the OmpK36 porin. (B) Top view of the OmpK36 porin. (C) Schematic representation of the OmpK36 protein sequence. Extracellular loop sequences from the 385 unique OmpK36 variants identified in Pathogenwatch were concatenated and clustered, resulting in 250 distinct concatenated loop variants. Extracellular loops and the constriction loop L3 are colored according to the color key; all other regions are shown in grey. The reference structure used for (A) and (B) is PDB entry 5O79 (https://www.rcsb.org/structure/5O79).

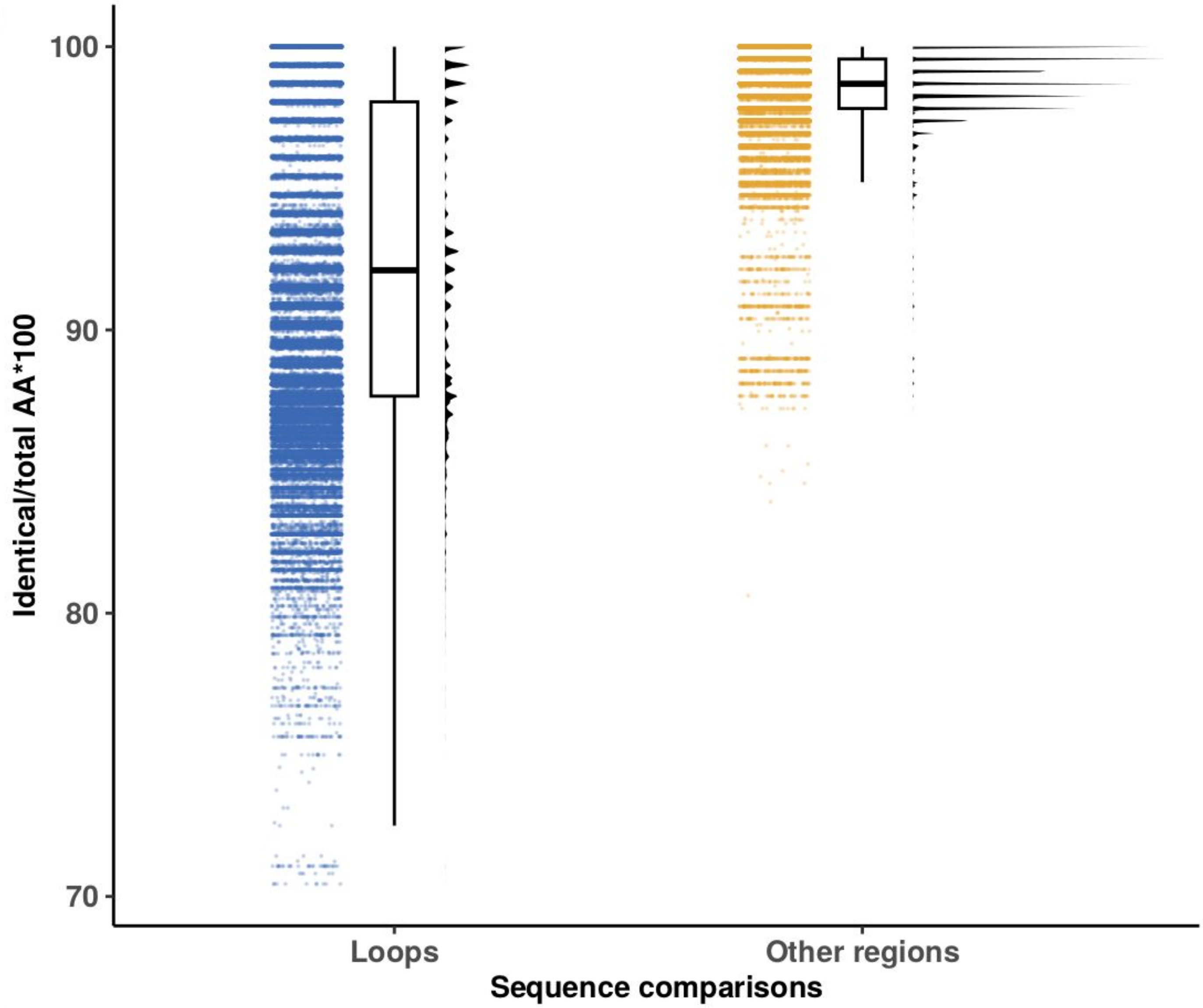

Figure S2. Protein identity between OmpK36 variants within loops and other regions. Individual points represent each pairwise comparison between variants considering the loop sequences (L1-L8) (blue) or other regions (yellow). For each comparison, the number of identical amino acids was divided by the length of the shortest sequence. The upper and lower limits of box-plots represent 75th and 25th quartile, the centre line represents the median and the whiskers extend to 1.5 × IQR. The right part of each plot represents the density plot obtained from the distribution of the comparison.

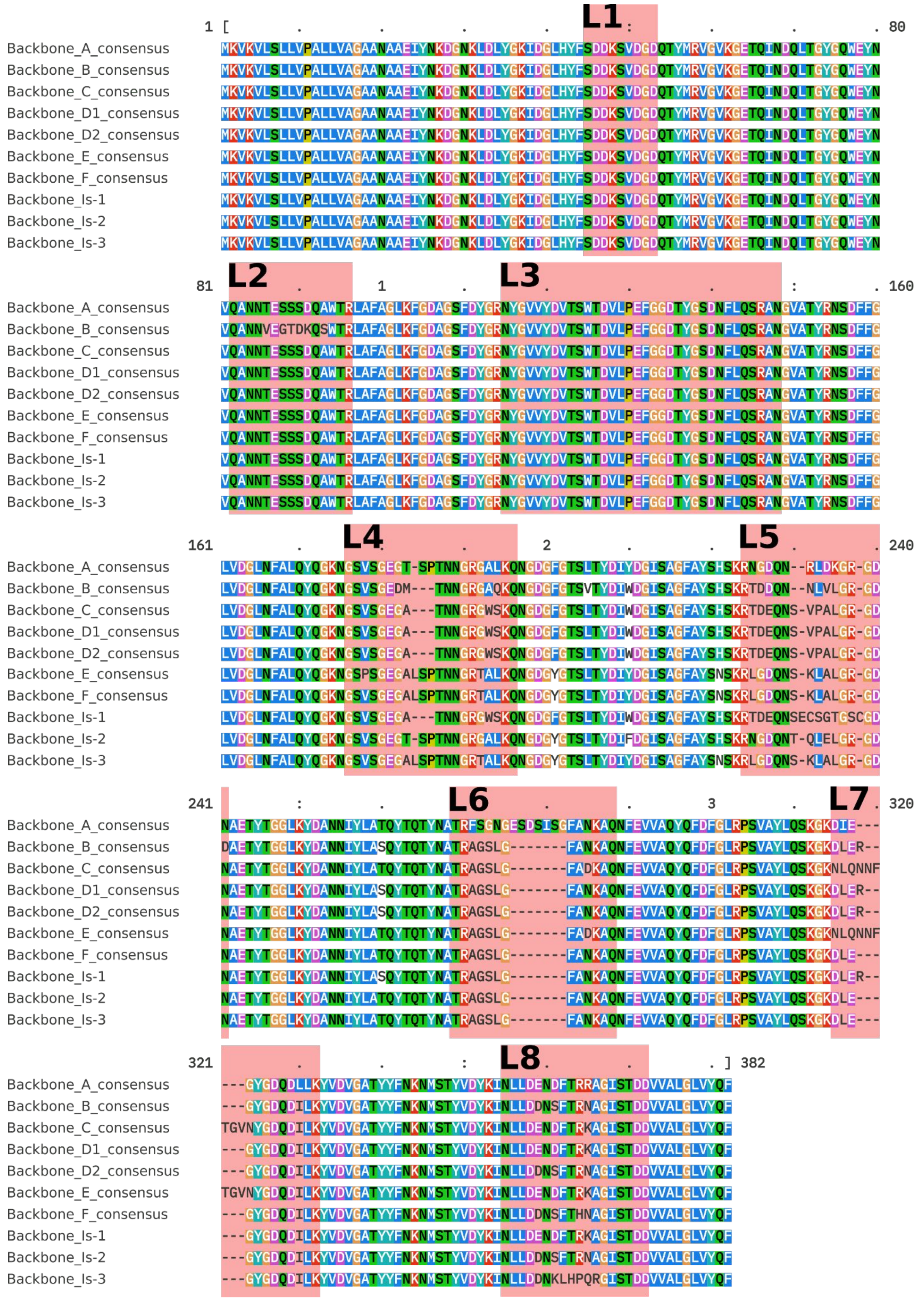

Figure S3. Alignment of consensus sequences of each OmpK36 backbone. Consensus sequences represent the most frequent amino acid at each position for each backbone. They were generated for each backbone using the function “cons” from EMBOSS v6.5.7.0 and visualized on MView. Amino acids are colored using the colormap “clustal” from MView which highlights amino acid physicochemical properties. For each loop, the number is indicated and the position is highlighted by a salmon background.

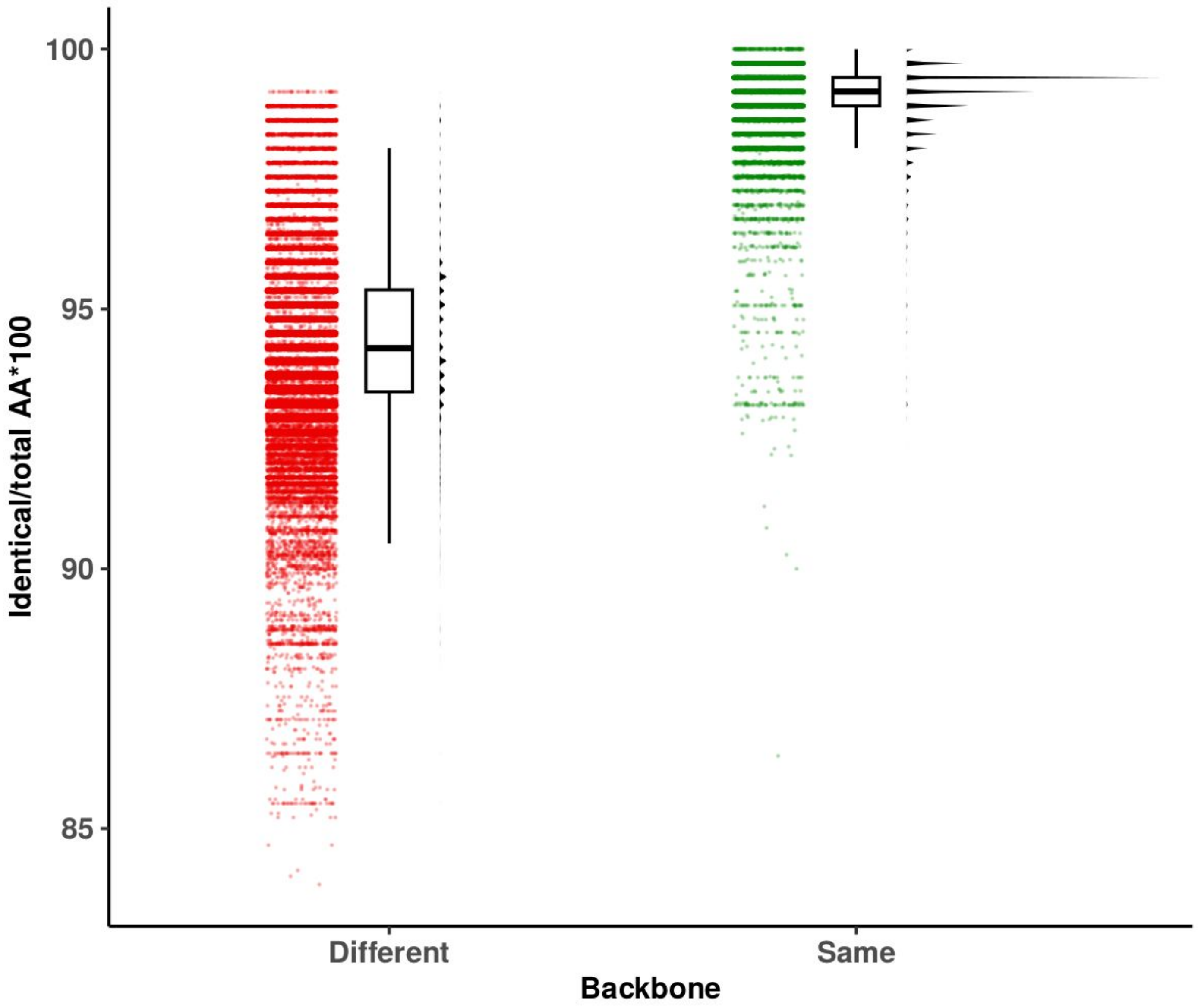

Figure S4. Pairwise protein identity between OmpK36 variants according to backbones. Individual points represent comparison of the whole OmpK36 sequence between variants belonging to different (red) or identical (green) backbones. For each comparison, the number of identical amino acids was divided by the length of the shortest sequence. The upper and lower limits of box-plots represent 75th and 25th quartile, the centre line represents the median and the whiskers extend to 1.5 × IQR. The right part of each plot represents the density plot obtained from the distribution of the comparison.

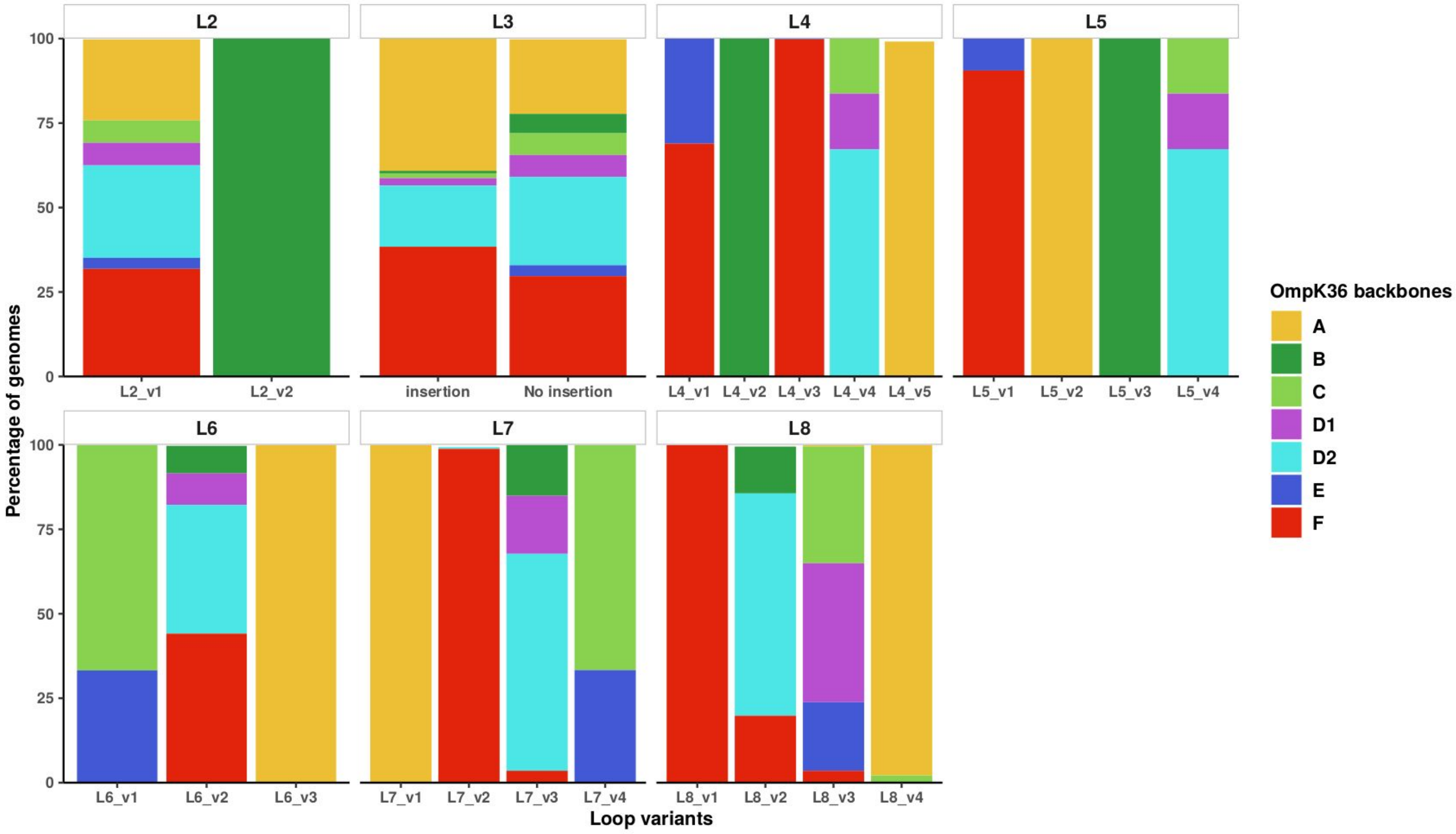

Figure S5. Variants of each individual OmpK36 loops and association with backbones. The backbones are colored according to the color key for each individual loop variant. The loop L1 is not represented because only one variant was observed. For the L3 constriction loop, we have separated the loops according to 'insertion' (GD, TD, D, SD or other) or 'absence of insertion' rather than according to variations over the whole loop.

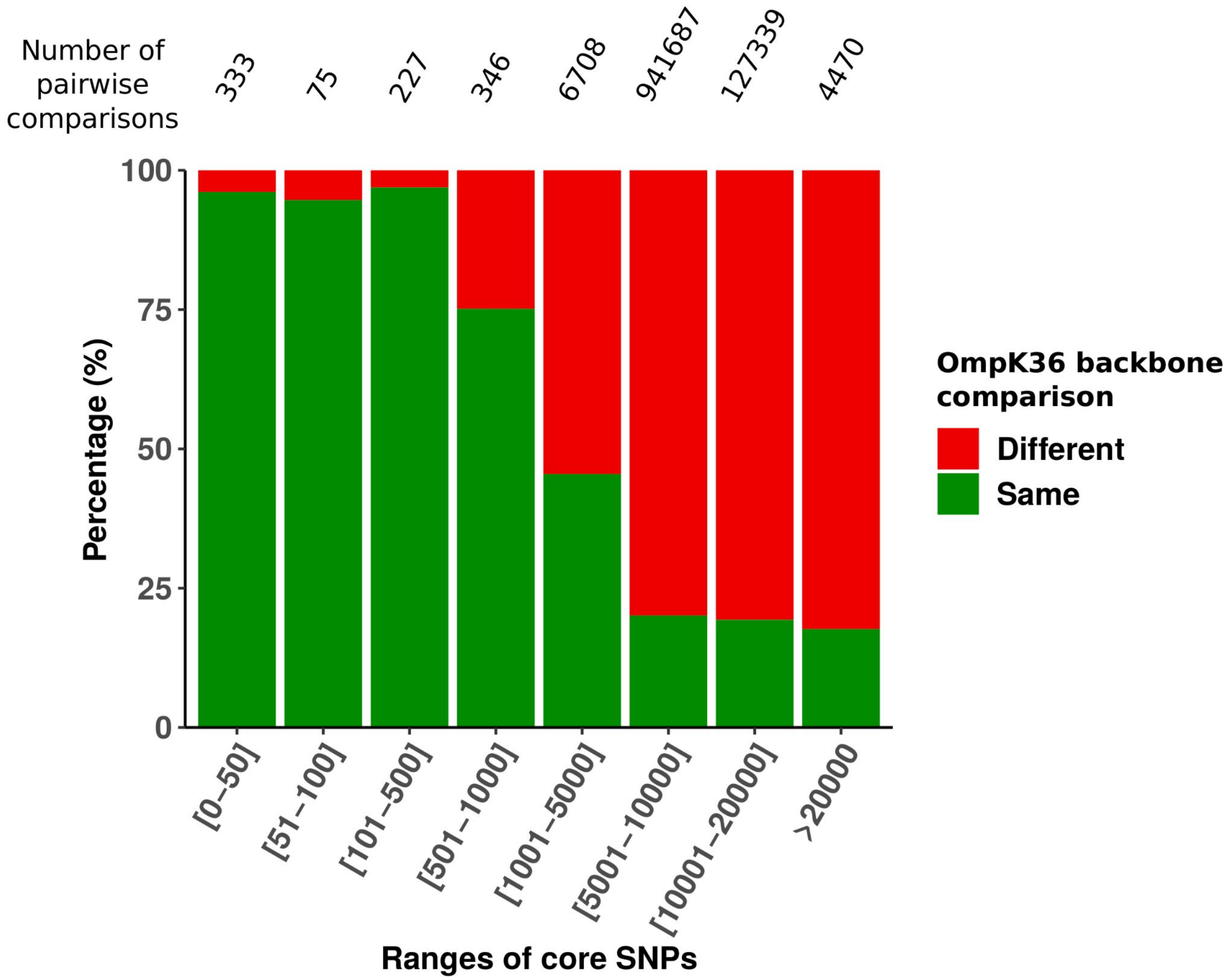

Figure S6. Percentage of genomes sharing different or identical OmpK36 backbones according to core SNPs. Core SNPs were computed from a core-genome alignment of 1471 genomes representative of the database diversity (“LIN 4 dataset”). The number of pairwise comparisons represented in each range of core SNPs is shown at the top of the bar chart. Pairwise comparisons between genomes carrying different or identical OmpK36 backbones are shown in red and green, respectively.

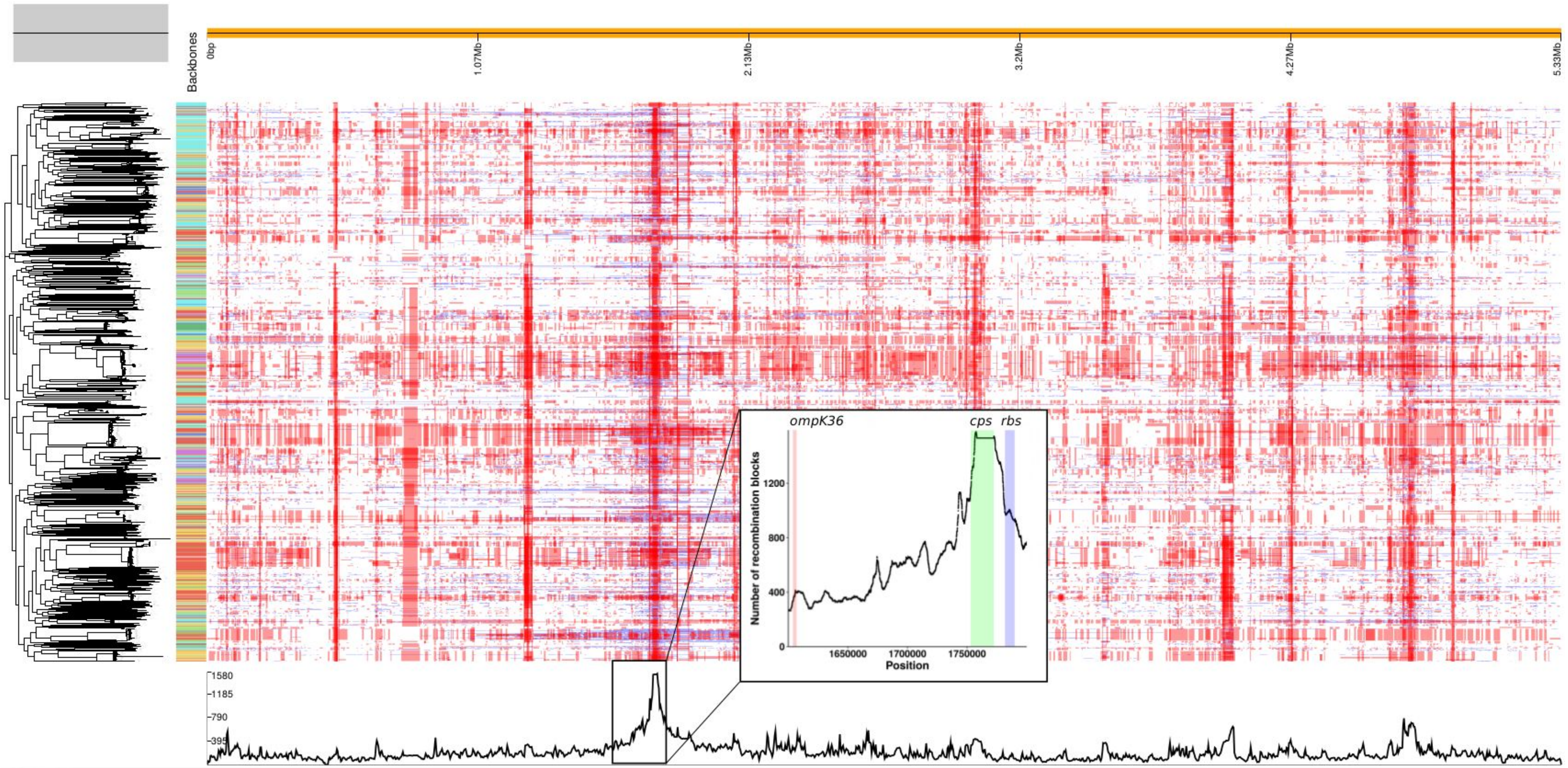

Figure S7. Predicted recombination events at the whole genome scale. Coordinates are shown on the x axis and refer to reverse positions in bp in the reference genome of *K. pneumoniae* HS11286 reference genome (RefSeq accession number: NC_016845.1). The number of recombination blocks identified using Gubbins is shown on the y axis. The OmpK36 backbones found in each genomes is shown in color on left part of the graph. The black box shows a zoom on the recombination hotspot corresponding to *rfb* (blue) and *cps* (green) operons in the vicinity of *ompK36* (salmon).

A

Host ID	Description
H1	*E. coli* MG1655
H2	*E. coli* MG1655 Δ*ompC*Δ*ompF*
H3	*E. coli* MG1655 Δ*ompC*Δ*ompF* + pACY_*ompK36*#A
H4	*E. coli* MG1655 Δ*ompC*Δ*ompF* + pACY_*ompK36*#F
H5	*E. coli* MG1655 Δ*ompC*Δ*ompF* + pACY_*ompK36*#D2
H6	*E. coli* MG1655 Δ*ompC*Δ*ompF* + pACY_*ompK36*#D1
H7	*E. coli* MG1655 Δ*ompC*Δ*ompF* + pACY_*ompK36*#B

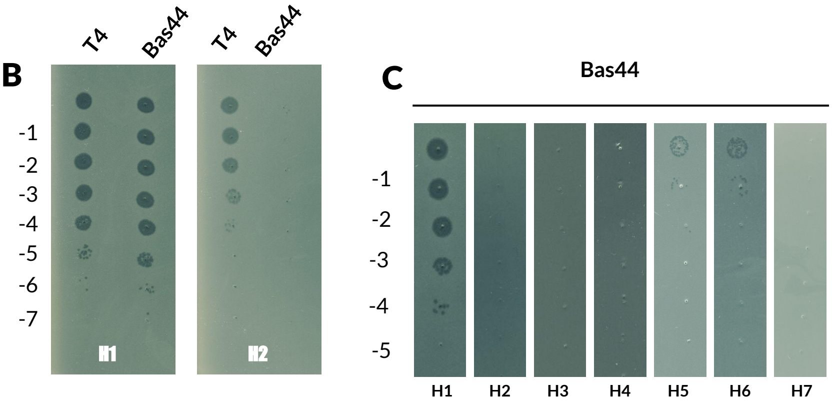

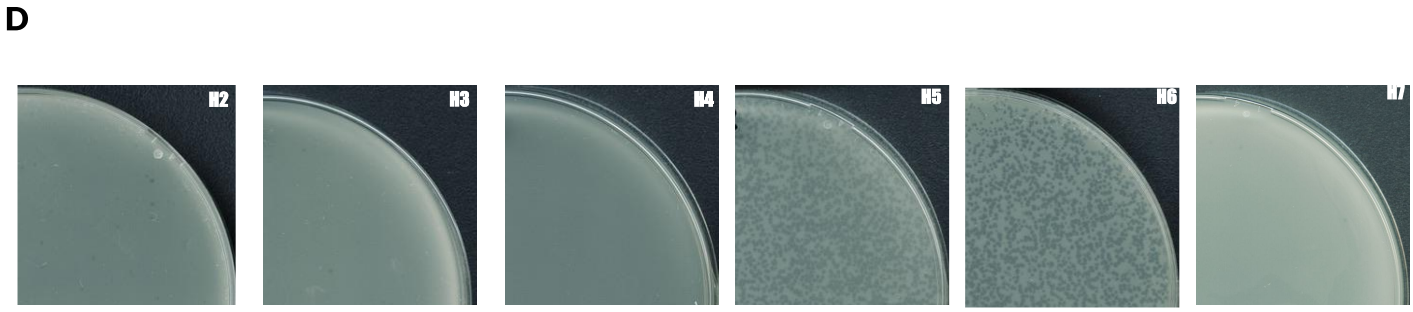

Figure S8. Isolation of Bas44 phage variants able to infect *E. coli* Δ*ompC*Δ*ompF* and expressing different OmpK36 porin variants. (**A**) Host strain numbering in this experiment. (**B, C**) Serial dilution of T4 or Bas44 lysates (log10 dilution factor) on bacterial lawns of host harboring different *ompK36* porin genes. (**D**) 50 μL of pure Bas44 lysate directly mixed with bacterial lawns.

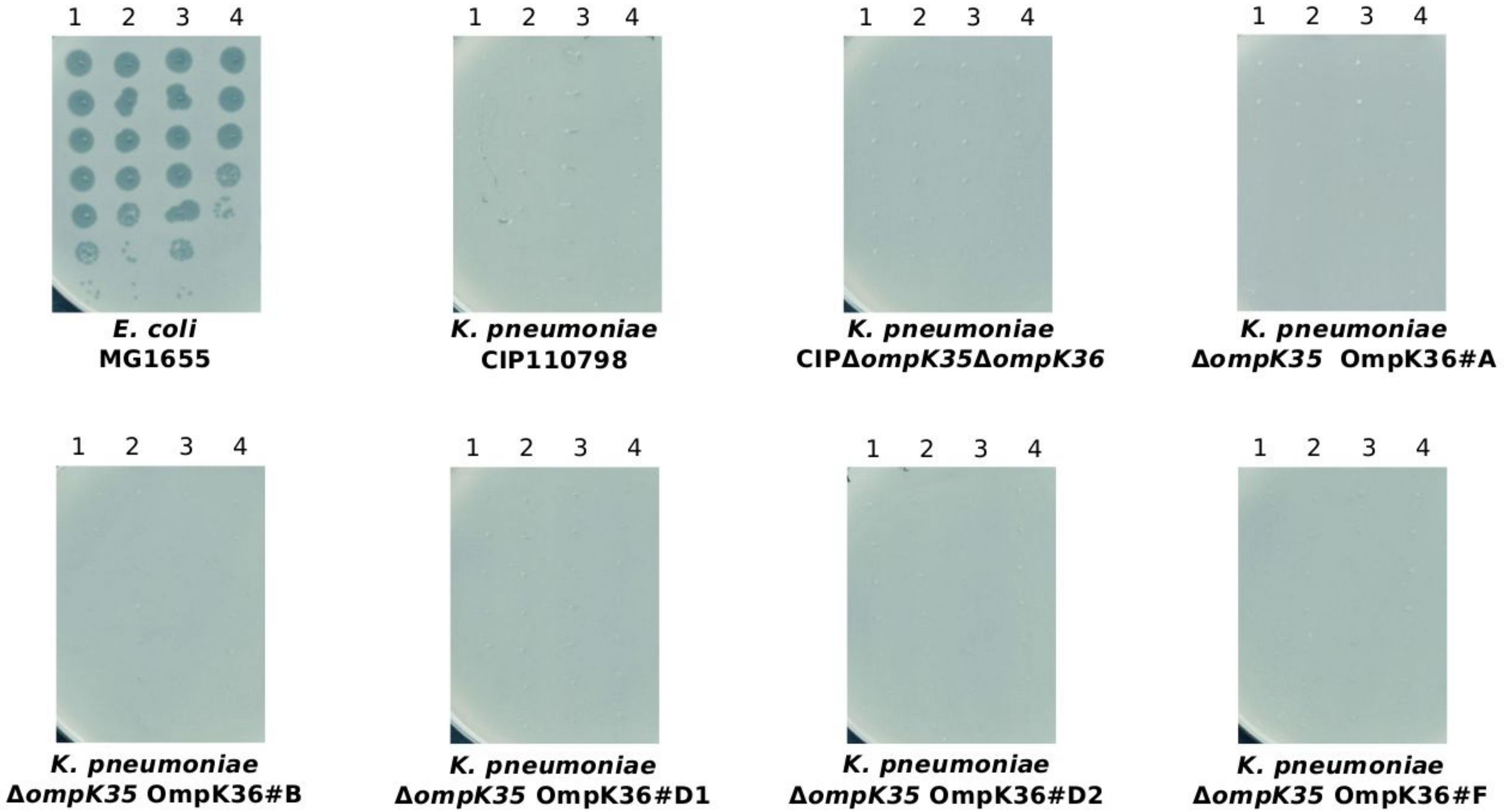

#	Phage lysate
1	T4
2	Bas44
3	Bas44<br>ompK36#D1 isolate<br>[0084_A978D]
4	Bas44<br>ompK36#D2 isolate<br>[0084_A978D, 0080_D100G]

Figure S9. Infection of *K. pneumoniae* CIP110798, CIPΔ*ompK35*Δ*ompK36* and mutants carrying the different OmpK36 porin variants with T4, Bas44 and its derivatives. Serial dilution of the phage lysates (log10 dilution factor) were applied on bacterial lawns of the different strains. Lysis plaque were obtained with *E. coli* MG1655, used as positive control. Neither *K. pneumoniae* CIP110798 nor any mutant exhibited susceptibility to the different phages tested.
