# Supplementary material for: Antibiotics and phages drive region-specific diversity of OmpK36 in Klebsiella pneumoniae
Source: mBio. 2025 Aug 18;16(9):e01343-25. doi: 10.1128/mbio.01343-25 (PMC12421954; doi:10.1128/mbio.01343-25)
Supplement: Supplemental methods — ompK36 mutant construction, construction of pACY plasmids carrying ompK36 representative, and Sanger sequencing-based competition assays. [file mbio.01343-25-s0002.docx]

**Supplementary Methods**

**Antibiotics and phages drive region-specific diversity of OmpK36 in *Klebsiella pneumoniae***

Guilhem Royer^1,2,3^, Raphaël Laurenceau^4^, Nicolas Cabanel^1^, Benjamin Bardiaux^5,6^, Cerdas-Mejías Karol Melissa^4^, David Bikard^4^, Isabelle Rosinski-Chupin^1^, Philippe Glaser^1^

1. Institut Pasteur, Université Paris Cité, CNRS UMR6047, Ecology and Evolution of Antibiotic Resistance Unit, Paris, France
2. Unité de Bactériologie, Département de Prévention, Diagnostic et Traitement des Infections, AP-HP, Hôpital Henri Mondor, Créteil 94000, France
3. EA 7380 Dynamyc, EnvA, UPEC, University of Paris-Est, Créteil 94000, France
4. Institut Pasteur, Université de Paris Cité, CNRS UMR3525, Synthetic Biology Unit, Paris, France
5. Institut Pasteur, Université Paris Cité, CNRS UMR3528, Structural Bioinformatics Unit, Paris, France
6. Institut Pasteur, Université Paris Cité, CNRS UMR3528, Bacterial Transmembrane Systems Unit, Paris, France

**o*mpK36* mutants construction**

Mutant strains of *K. pneumoniae* CIP110798 deleted for *ompK35* and carrying *ompK36* variants representative of the different backbones were constructed as follows.

To build plasmid pACYZeoFRT_2, the *zeoR* (zeocin resistance) gene was amplified using *Bsa*I-sites containing primers Zeo_F/R (Table S10). FLP recognition target (FRT) sites containing *Bsa*I on one side and *Hind*III (FRT1) or *BamH*I (FRT2) on the other side were then ligated with *zeoR* and pACYt previously digested to build the vector pACYZeoFRT_2. From this plasmid, we amplified the sequence FRT1-zeoR-FRT2 with primers ompK35zeo_F/R using Q5 high fidelity DNA polymerase (New England Biolabs). *Dpn*I-digested PCR products were electroporated into *K. pneumoniae* CIP110798 strain previously electroporated with the plasmid p15Red, encoding the lambda Red recombinase to perform *ompK35* deletion through homologous recombination as previously described [(1)](https://www.zotero.org/google-docs/?2Yk3ck). In the same way, the sequence from FRT-neoR-tse2-FRT was amplified from a modified version of pSLC-246 (Addgene) using primers ompK36_neo-tse2_F/R. This fragment was used to delete *ompK36* from CIP110798Δ*ompK35* through homologous recombination leading to strain CIP110798Δ*ompK35*Δ*ompK36*FRT. Lastly, we recombined FRT sites by induction of the flippase from electroporated plasmid p15Flip to get the mutant strain CIP110798Δ*ompK35*Δ*ompK36* (designated hereafter Δ*ompK35*Δo*mpK36*).

In parallel, we amplified *ompk36* variants representative of the main OmpK36 porin backbones (A, B, D1, D2 and F) using DNA from isolates carrying these alleles and primers ompK36_replace_F/R and AmpliTaq Gold DNA polymerase (ThermoFisher Scientific). The PCR products were electroporated into CIP110798Δ*ompK35*Δ*ompK36*FRT to perform homologous recombination into the original location of *ompK36*. Recombinants were counter-selected by induction of the *tse2* toxin-coding gene with rhamnose 0.2%. Finally, we recombined FRT sites by induction of the flippase to get the following mutant strains: CIP110798Δ*ompK35*Δ*ompK36*::*ompK36*-backboneA, B, D1, D2 and F (designated hereafter OmpK36#A, OmpK36#B, OmpK36#D1, OmpK36#D2 and OmpK36#F).

All mutants were controlled by PCR (Table S10) and were fully sequenced by Illumina 2*150 pb (NEBNext Ultra II FS DNA library prep kit, Illumina) on Illumina Novaseq 6000 after DNA extraction using Blood and Tissue DNA easy kit (Qiagen). Mutant genomes were compared with the original strain using Breseq [(2)](https://www.zotero.org/google-docs/?EjTCkS) with standard parameters. Sequences are available in the Bioproject PRJEB88512.

**Construction of pACY plasmids carrying *ompK36* representative**

We constructed plasmids using the same *ompK36* variants representative of the main OmpK36 porin backbones (A, B, D1, D2, and F) as described above. The different *ompK36* variants were amplified from genomic DNA of isolates carrying the corresponding alleles, using AmpliTaq Gold DNA polymerase (ThermoFisher Scientific) and primers XbaOmpK36_F and HindIIIompK36_R to introduce restriction sites flanking the amplicons (Table S10). For each *ompK36* variant, the XbaI- and HindIII-digested pACY plasmid and *ompK36* amplicon were ligated to generate a pACY plasmid carrying the corresponding variant. The resulting plasmids were then transformed into XL1-Blue Competent Cells (Agilent) and subsequently purified using a miniprep kit (Macherey-Nagel). Purified plasmids were used to complement an *E. coli* K-12 MG1655 Δ*ompC*Δ*ompF* strain *in trans*, to assess phage host range.

**Sanger sequencing-based competition assays**

To compare mutant growth in competition assay, we took advantage of polymorphisms in *ompK36* sequence among the five alleles and using Sanger sequencing as described by Thiriet-Rupert et al. [(3)](https://www.zotero.org/google-docs/?1Kxfk4). Briefly, the strains were grown until stationary phase, diluted to reach OD600nm of 0.1 and then cultured overnight (ON) after diluting this solution 1/200 in LB Miller. We mixed these ON cultures at a 1:1 ratio, combining all possible pairs of strains (n=10). We sampled 100 µL of these pairs to get a reference ratio at time T0. Then, the strain mixtures were diluted to approximately 10^6^ CFU/mL in LB Miller or MHB + 0.006 µg/mL ertapenem and incubated at 37°C with continuous shaking. These cultures were diluted 1/200 twice a day in the fresh corresponding medium (LB Miller or MHB + ertapenem respectively) for 3 days. The fourth day we performed subcultures in a 24-well plate (TPP) in the corresponding media until reaching the beginning of the stationary phase and sampled them as for T0 in order to obtain Tf (final T) samples.

The ratio of the two strains was determined by comparing the amplitude of Sanger sequencing peaks of polymorphic site following PCR amplification using primer matching identical sequences between the two tested strains (OmpK36_4_F/OmpK36_2_R or OmpK36for/OmpK36_1_R). The chromatograms were analyzed using sangerseqr package [(4)](https://www.zotero.org/google-docs/?BuGQD0). We corrected ratios using the T0 data as follows: ratio=(peak amplitude strain A Tf/peak amplitude strain B Tf)/(peak amplitude strain A T0/peak amplitude strain B T0). Finally, we computed the mean and standard deviation of the log transformed average ratio obtained from three independent competition assays for each pair in each condition and compared it to the value 0 (i.e. no difference between strains) using one sample t-test.

**References**

[1. Datsenko KA, Wanner BL. 2000. One-step inactivation of chromosomal genes in *Escherichia coli* K-12 using PCR products. Proc Natl Acad Sci U S A 97:6640–6645.](https://www.zotero.org/google-docs/?a7pnY4)

[2. Barrick JE, Colburn G, Deatherage DE, Traverse CC, Strand MD, Borges JJ, Knoester DB, Reba A, Meyer AG. 2014. Identifying structural variation in haploid microbial genomes from short-read resequencing data using breseq. BMC Genomics 15:1039.](https://www.zotero.org/google-docs/?a7pnY4)

[3. Thiriet-Rupert S, Josse J, Perez-Pascual D, Tasse J, Andre C, Abad L, Lebeaux D, Ghigo J-M, Laurent F, Beloin C. 2023. Analysis of in-patient evolution of *Escherichia coli* reveals potential links to relapse of bone and joint infections. J Infect Dis jiad528.](https://www.zotero.org/google-docs/?a7pnY4)

[4. sangerseqR: Tools for Sanger Sequencing Data in R version 1.26.0 from Bioconductor. https://rdrr.io/bioc/sangerseqR/. Retrieved 29 November 2023.](https://www.zotero.org/google-docs/?a7pnY4)
